# The insulin-like peptide INS-27 mediates a muscle-to-neuron feedback signal coupling muscle activity with AMPA receptor trafficking

**DOI:** 10.1371/journal.pgen.1011786

**Published:** 2025-07-18

**Authors:** Bethany J. Rennich, Regina M. Powers, Samantha Moores, Molly Hodul, Peter Juo

**Affiliations:** 1 Department of Developmental, Molecular and Chemical Biology, Tufts University School of Medicine, Boston, Massachusetts, United States of America; 2 Graduate Program in Neuroscience, Graduate School of Biomedical Sciences, Tufts University School of Medicine, Boston, Massachusetts, United States of America; 3 Graduate Program in Genetics, Molecular and Cellular Biology, Graduate School of Biomedical Sciences, Tufts University School of Medicine, Boston, Massachusetts, United States of America; University of California San Francisco, UNITED STATES OF AMERICA

## Abstract

Regulation of AMPA Receptor (AMPAR) levels at synapses controls synaptic strength and is a major mechanism underlying learning and memory. Growing evidence indicates that AMPAR trafficking can be regulated by extracellular factors. Here, we show that the insulin-like peptide INS-27 mediates a muscle-to-neuron signal that promotes surface levels of the *C. elegans* AMPAR GLR-1 at synapses in pre-motor AVA interneurons that reside two synaptic layers upstream of the neuromuscular junction. Mutants lacking cholinergic neuromuscular signaling or muscle activity trigger an increase in surface GLR-1 levels in upstream AVA neurons. Genetic data suggest that this signal is dependent on the dense-core vesicle regulator *unc-31*/CAPS, the insulin-like peptide INS-27, which is one of the most highly expressed neuropeptides in muscle, and the Insulin/IGF-1 receptor DAF-2. *ins-27* loss-of-function mutants exhibit decreased surface GLR-1 levels and defects in glutamatergic behavior. Further, loss of neuromuscular junction signaling stimulates secretion of INS-27 from muscle in an *unc-31*/CAPS-dependent manner. Our data support a model in which INS-27 is released from muscle and signals via DAF-2/Insulin/IGF-1 receptors to promote surface levels of GLR-1 in AVA neurons. Our study reveals a potential feedback signal that couples muscle activity with surface AMPARs in upstream neurons.

## Author summary

Glutamate is a widely used neurotransmitter in the brain that is released from presynaptic neurons and detected by glutamate receptors on downstream postsynaptic neurons. Changes in glutamate receptor levels at the postsynaptic cell surface is highly regulated and mediates learning and memory. Although much research has identified the molecular steps that act inside neurons to regulate the number of glutamate receptors at synapses, less is known about external signals that regulate glutamate receptors at synapses, especially by signals that originate from non-neuronal cells. We discovered that loss of neuromuscular junction signaling or loss of muscle activity triggers a feedback pathway in the nematode *C. elegans* that leads to increased levels of glutamate receptors at the postsynaptic surface of upstream neurons. This feedback pathway is dependent on a gene involved in release of neuropeptides, UNC-31, an insulin-like peptide called INS-27 and the DAF-2 insulin receptor. Our data support a model where loss of muscle activity promotes release of INS-27 from muscle leading to increased surface levels of glutamate receptors in neurons via DAF-2 insulin receptors. This study reveals a potential feedback pathway that couples muscle activity with glutamate receptor levels at synapses upstream in a circuit that controls locomotion.

## Introduction

Regulation of AMPA-type glutamate receptor (AMPAR) levels and function in the postsynaptic membrane controls synaptic strength and is a principal mechanism underlying information processing and synaptic plasticity in the brain [1]. Great progress has been made identifying transmembrane auxiliary subunits and intracellular proteins that interact with AMPARs to regulate their trafficking, anchoring and function at synapses [2,3]. In addition, a growing number of extracellular, secreted factors have emerged that can control AMPAR trafficking and clustering [4,5]. Some secreted factors, such as Neuronal Pentraxins [6,7] and Noelins [8,9], interact directly with the N-terminal domain of AMPAR subunits and function as extracellular scaffolds that cluster and anchor AMPARs. Other factors such as TNFα [10,11], BDNF [12], Netrin [13], and Wg/Wnt [14], are diffusible ligands that engage intracellular signaling pathways via their cognate receptors to indirectly control AMPAR trafficking. Although some of these factors are secreted from neurons, others, including glypicans [15,16] and Chordlin-like-1 [17] are secreted from glia [18]. However, our understanding of AMPAR-regulatory factors that are secreted from other non-neuronal cells and tissues, especially those that act at a distance, is limited.

Evidence from model organisms suggest that many signals from distal tissues impinge on the brain. Recent proteomic studies from *Drosophila* have identified hundreds of secreted factors potentially involved in inter-tissue and inter-organ communication, including factors that may mediate signaling between peripheral tissues and the brain [19,20]. In *C. elegans*, several neuropeptides have been identified that mediate signaling between the intestine and the nervous system. For example, the intestine secretes the neuropeptide-like peptide NLP-40 to regulate defecation [21,22], synapse development [23], and neuron regeneration [24]. In response to food status the intestine secretes the insulin-like peptides (ILPs) INS-1 and INS-31 to modulate sensory neuron function [25,26] and INS-7 to control fat storage [27]. The intestine also releases INS-11 to regulate aversive learning behavior [28].

During exercise, muscle is known to secrete growth factors, cytokines, and peptides (termed myokines), that signal in an autocrine manner to muscle as well as to other more distal tissues [29]. Over 600 factors are secreted from cultured human myocytes [30] and *in vivo* proteomics identified at least 50 factors that are secreted from muscle in *Drosophila* [19]. At the vertebrate and invertebrate neuromuscular junction (NMJ), muscle utilizes retrograde signals to presynaptic motor neurons to coordinate synapse development and alter synaptic strength [31,32]. Some of these signals are mediated by synaptic cell adhesion molecules [33–38], whereas others are mediated by secreted factors [39,40]. Interestingly, in *C. elegans*, the calcium binding DCV regulator, MUNC-13b/AEX-1 [41], and the neuropeptide processing enzyme AEX-5 mediate a retrograde signal at the NMJ [42], suggesting that muscle can release neuropeptides from DCVs to communicate with the nervous system. These studies reveal that muscle can secrete factors that act locally across one synaptic layer to regulate presynaptic motor neuron development and function. Intriguingly, the known beneficial effects of muscle contraction during exercise on metabolism and brain function in mammals may be mediated by myokines that act on distal tissues [29,43,44]. For example, in rodent models, muscle-released Cathepsin B has been implicated in regulating neurogenesis in the brain via BDNF [45], and muscle-released Irisin appears to mediate many of the beneficial effects of exercise on metabolism and cognition [46–48]. However, the function of the vast majority of muscle-secreted factors and, in particular, the specific mechanisms by which they impact the nervous system, are incompletely understood.

**Fig 1:**
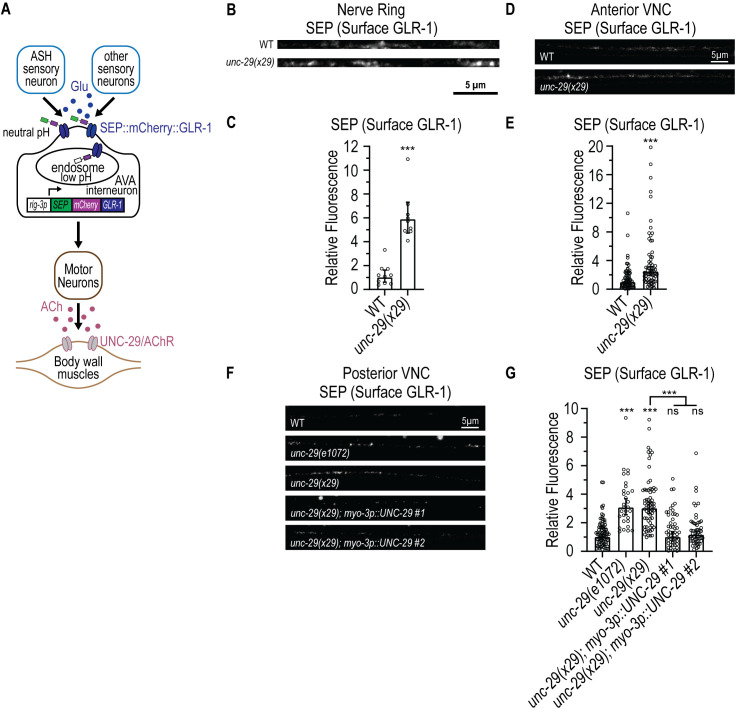
Loss of cholinergic NMJ signaling results in increased surface GLR-1 levels in upstream AVA pre-motor interneurons. (**A)** Illustration of the ASH-AVA-MN-muscle circuit. Stimulation of glutamatergic sensory neurons such as ASH leads to activation of AVA pre-motor interneurons via the AMPA receptor GLR-1 and downstream cholinergic motor neurons. Release of ACh from motor neurons at the NMJ leads to contraction of body wall muscle and backward movement away from the stimulus. Levels of GLR-1 at the neuronal surface in AVA neurons can be monitored using GLR-1 tagged with Superecliptic pHluorin (SEP) and mCherry. The *rig-3* promoter drives expression of SEP::mCherry::GLR-1 specifically in AVA neurons. The pH sensitive SEP fluoresces at neutral pH at the synaptic surface but is quenched in acidic internal compartments, whereas mCherry fluoresces both at the cell surface and in endosomes. Thus, the SEP fluorescence signal represents surface GLR-1 whereas the mCherry fluorescence signal represents total GLR-1. **(B)** Representative images showing SEP fluorescence (surface GLR-1) in AVA nerve ring (NR) processes (straightened) in animals expressing a SEP::mCherry::GLR-1 integrated transgene (*akIs201*) in Wild-type (WT) or *unc-29(x29)* mutant animals. **(C)** Quantification of SEP fluorescence (Normalized) for the strains shown in **B.** The graph shows Geometric mean, lower 95% Confidence Interval (CI) - upper 95% CI for WT (1.00, 0.61-1.63 n = 10) and *unc-29(x29)*(5.88, 4.73-7.32, n = 9) animals. Values that differ significantly from WT (Kolmogorov-Smirnov test) are indicated as follows: ***p < 0.001. **(D)** Representative images showing SEP fluorescence (surface GLR-1) in the AVA processes in the anterior VNC in animals expressing a SEP::mCherry::GLR-1 integrated transgene (*akIs201*) in WT or *unc-29(x29)* mutants. **(E)** Quantification of SEP fluorescence (Normalized) for the strains shown in **D.** The graph shows Geometric mean, lower 95% CI - upper 95% CI for WT (1.00, 0.77-1.30, n = 65) and *unc-29(x29)* animals (2.48, 1.94-3.18, n = 64). Values that differ significantly from WT (Kolmogorov-Smirnov test) are indicated as follows: ***p < 0.001. (F) Representative images showing SEP fluorescence (surface GLR-1) in the AVA processes in the posterior VNC in animals expressing a SEP::mCherry::GLR-1 integrated transgene (*akIs201*) in WT, *unc-29(e1072),* or *unc-29(x29)* mutants, or *unc-29(x29)* mutants expressing WT *unc-29* cDNA under control of the *myo-3* promoter *(pzEx476: myo-3p::UNC-29 line #1* and *pzEx477: myo-3p::UNC-29 line #2*). (G) Quantification of SEP fluorescence (Normalized) for the strains shown in F. Graphs show Geometric mean, lower 95% CI - upper 95% CI for WT (1.00, 0.84-1.19, n = 83), *unc-29(e1072)* (3.07, 2.56-3.68, n = 30), *unc-29(x29)* (3.02, 2.64-3.45, n = 66), *unc-29(x29); pzEx476 (myo-3p::UNC-29 line 1)*(1.00, 0.72-1.38, n = 51), and *unc-29(x29); pzEx477 (myo-3p::UNC-29 line 2)* (1.16, 0.94-1.42, n = 54) animals. Values that differ significantly from WT (Kruskal-Wallis, Dunn’s multiple comparison test) are shown above each bar. Other comparisons are marked by brackets. ns, p > 0.05. ***p < 0.001.

We previously discovered that the *C. elegans* homolog of Vascular Endothelial Growth Factor (VEGF), PVF-1, is secreted from muscle to promote cell surface levels of the AMPA-type glutamate receptor GLR-1 at synapses in AVA interneurons [49]. AVA interneurons are one of five pairs of GLR-1 expressing pre-motor command interneurons that receive and integrate synaptic input from upstream glutamatergic sensory neurons to coordinate locomotion [50–53]. Aversive stimuli (i.e., mechanical touch or chemical repellants) detected by the glutamatergic sensory neuron ASH leads to activation of AVA to direct backward locomotion away from the stimuli (see Fig 1A for circuit diagram) [51,52,54–58]. We previously found that loss of *pvf-1*/VEGF or the VEGF receptor homologs, *ver-1* or *ver-4,* results in defects in a glutamatergic mechanosensory behavior mediated by ASH called the nose-touch response [49]. *pvf-1*, *ver-1* or *ver-4* mutants also exhibit decreased levels of GLR-1 at the cell surface at synapses in AVA neurons. Rescue experiments suggest that the VERs act in AVA neurons to mediate the effects of muscle-secreted PVF-1 [49], leading to an intriguing model whereby PVF-1 is released from muscle to regulate surface levels of GLR-1 at synapses that reside two synaptic layers upstream of the NMJ. These results prompted us to investigate whether NMJ signaling or muscle function might regulate AMPARs in upstream neurons.

Here, we find that loss of NMJ signaling or muscle activity triggers a potential compensatory feedback pathway that regulates AMPARs in upstream AVA neurons. This feedback pathway is dependent on the DCV regulator *unc-31*/Calcium-Activated Protein for Secretion (CAPS), but unexpectedly, does not require PVF-1. Instead, we find that the feedback pathway is dependent on the insulin-like peptide INS-27, which is one of the most highly-expressed neuropeptides in muscle, and the Insulin/IGF-1 receptor, DAF-2. We also show that loss of NMJ signaling leads to increased secretion of INS-27 from muscle in an *unc-31*/CAPS-dependent manner. This study identifies a function for INS-27 by showing that it is secreted from muscle to promote cell surface levels of AMPARs in upstream neurons.

## Results

### Loss of NMJ signaling results in increased surface GLR-1 levels in upstream AVA interneurons

Our previous study showed that a factor secreted from muscle could promote surface levels of GLR-1/AMPARs at synapses in upstream AVA interneurons [49]. Why would muscle release a factor that regulates GLR-1 in upstream neurons? We hypothesized that reduced NMJ signaling could trigger a feedback pathway that drives a compensatory increase in surface levels of GLR-1 in AVA interneurons. We tested this idea by measuring surface levels of GLR-1 in AVA neurons in animals lacking NMJ signaling. We monitored total and surface levels of GLR-1 with a transgenic strain (*akIs201*) that expresses a functional GLR-1 tagged at the N-terminus with the pH-sensitive GFP variant, superecliptic pHluorin (SEP), and mCherry (SEP::mCherry::GLR-1) in AVA neurons (using the *rig-3* promoter), as previously described [49,59–61]. SEP fluoresces at the cell surface in the neutral pH of the extracellular space but is quenched in acidic internal compartments. Thus, quantification of SEP fluorescence provides an estimate of GLR-1 on the cell surface. mCherry has a lower pKa and fluoresces both at the cell surface and in internal compartments providing an estimate of total GLR-1 abundance (Fig 1A) [59].

In order to test whether loss of NMJ signaling could affect surface levels of GLR-1 in AVA neurons, we measured surface levels of GLR-1 in mutants lacking *unc-29*, which is a non-alpha AChR subunit that functions at the NMJ [62]. GLR-1 localizes to synaptic puncta in the nerve ring and ventral nerve cord (VNC) [59,60,63–65]. Interestingly, loss of *unc-29(x29)*/AChR triggered a ~ 2–6-fold increase in SEP fluorescence (surface GLR-1 levels) at synapses globally throughout AVA neurons: in the nerve ring (Fig 1B and 1C), the anterior VNC (Fig 1D and 1E) and the posterior VNC (Fig 1F and 1G). This effect could be rescued by expression of wild-type *unc-29* cDNA specifically in body wall muscle using the *myo-3* promoter (Fig 1F and 1G). Additionally, we observed a similar increase in surface GLR-1 levels in another independent loss-of-function mutant allele of *unc-29(e1072)*(Fig 1F and 1G) and in mutants lacking *unc-38,* which is an alpha AChR subunit that also functions at the NMJ (S1A and S1B Fig) [62,66]. We also tested whether loss of NMJ signaling affected NMDA receptors in AVA neurons by measuring surface levels of the NMDA receptor subunit NMR-2 in *unc-29*/AChR mutants using a transgene (*akIs237*) expressing SEP and mCherry-tagged NMR-2 in AVA neurons (S2A Fig) [67]. We found no significant increase in surface levels of NMR-2 (SEP fluorescence)(p = 0.7) in the posterior VNC of AVA neurons in *unc-29*/AChR mutants (S2B–S2C Fig), suggesting that the effect of loss of NMJ signaling on GLR-1 may be specific. Together, these data indicate that defective cholinergic signaling at the NMJ results in a global increase in surface GLR-1 levels at synapses throughout AVA processes, revealing a potential feedback pathway that couples NMJ activity with GLR-1 surface levels in upstream AVA pre-motor interneurons.

Whereas surface GLR-1 levels are consistently and robustly increased throughout AVA neurons in several AChR subunit mutant alleles, changes in mCherry fluorescence (Total GLR-1) were more variable (S1C–S1H Fig). For example, although we observed a comparable increase in surface GLR-1 in two independent *unc-29* mutant alleles (*e1072* and *x29*)(Fig 1F and 1G), total GLR-1 levels were only increased in the *x29* allele (S1G–S1H Fig), suggesting that the effects on surface and total GLR-1 are uncoupled. Nevertheless, because the SEP::mCherry::GLR-1 transgene is under control of the AVA neuron-specific promoter *rig-3*, we tested if the increase in total GLR-1 in *unc-29(x29)* mutants might be due to increased *rig-3* promoter activity. We found no change in *rig-3* promoter activity in *unc-29(x29)* mutants using a transcriptional reporter (*rig-3p::NLS-GFP-LacZ*)(S3 Fig). This data suggests that the increase in total GLR-1 in *unc-29(x29)* is not due to increased *rig-3* promoter activity and is likely due to a post-transcriptional mechanism. For the remainder of this study, we focused our analysis on surface levels of GLR-1 since this is the pool of receptors most relevant for GLR-1 function and the effects of the feedback pathway on SEP fluorescence were robust.

### Acute loss of muscle activity is sufficient to induce increased surface GLR-1 in AVA interneurons

Our data show that chronic loss of NMJ signaling is sufficient to trigger an increase in surface GLR-1 levels in AVA interneurons. This finding raises several questions. First, we wondered whether the feedback pathway was being triggered by the loss of NMJ signaling or by the subsequent loss of muscle function. Second, given the chronic nature of the NMJ mutants that we used, we wondered whether the increase in surface GLR-1 was due to an indirect effect on development or if the feedback pathway could be triggered in developed animals. To address these questions, we measured surface GLR-1 levels after acutely inactivating muscle using two independent conditional mutants and a chemical-genetic approach.

**Fig 2:**
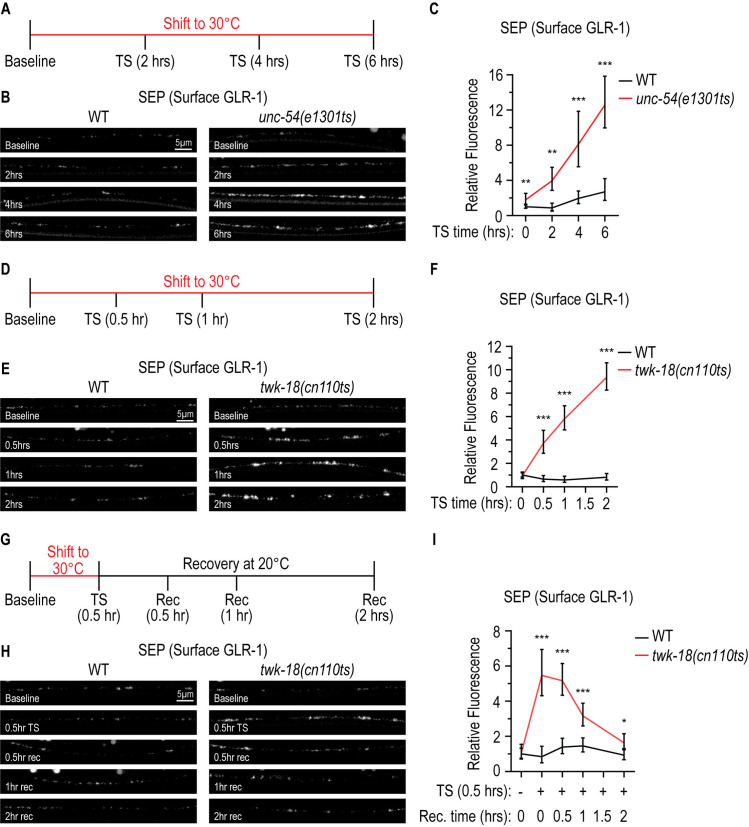
Acute loss of muscle activity is sufficient to increase surface GLR-1 levels in AVA pre-motor interneurons. (**A)** Timeline illustrating the temperature shift (TS) paradigm used for *unc-54(e1301ts)* mutants. Larval stage 4 (L4) worms were imaged before (baseline, TS = 0 hours) or after a TS to 30°C for 2, 4, or 6 hours (hrs). **(B)** Representative images showing SEP fluorescence (surface GLR-1) in the AVA processes in the posterior VNC in animals expressing a SEP::mCherry::GLR-1 integrated transgene (*akIs201*) in WT and *unc-54(e1301ts)* temperature-sensitive mutants at baseline and after 2, 4 or 6 hrs TS. **(C)** Quantification of SEP fluorescence (surface GLR-1) at each imaging timepoint for strains shown in **B.** The graph shows Geometric mean, lower 95% CI - upper 95% CI for WT (baseline (Norm.): 1.00, 0.83-1.21, n = 40; 2-hr TS: 0.88, 0.55-1.41, n = 32; 4-hr TS: 1.95, 1.37-2.79, n = 26; 6-hr TS: 2.70, 1.73-4.21, n = 27) and *unc-54(e1301ts)* (baseline: 1.80, 1.28-2.53, n = 33; 2-hr TS: 3.96, 2.86-5.49, n = 25; 4-hr TS: 8.11, 5.55-11.86, n = 17; 6-hr TS: 12.57, 9.97-15.85, n = 23). Values that differ significantly from WT (Kolmogorov-Smirnov test) are shown above each data point. **p < 0.01, ***p < 0.001. **(D)** Timeline illustrating the TS paradigm used for *twk-18(cn110ts)*. L4 worms were imaged before (baseline, TS = 0 hrs) or after a TS to 30°C for 0.5, 1, or 2 hrs. **(E)** Representative images showing SEP fluorescence (surface GLR-1) in the AVA processes in the posterior VNC in animals expressing a SEP::mCherry::GLR-1 integrated transgene (*akIs201*) in WT and *twk-18(cn110ts)* temperature-sensitive mutants at baseline and after 0.5, 1 or 2 hrs TS. **(F)** Quantification of SEP fluorescence (surface GLR-1) at each imaging timepoint for strains shown in **E.** The graph shows Geometric mean, lower 95% CI - upper 95% CI for WT (baseline (Norm.): 1.00, 0.84-1.19, n = 45; 0.5-hr TS: 0.66, 0.45-0.96, n = 31; 1-hr TS: 0.59, 0.38-0.90, n = 12; 2-hr TS: 0.81, 0.58-1.14, n = 19) and *twk-18(cn110ts)* (baseline: 0.94, 0.70-1.26, n = 39; 0.5-hr TS: 3.72, 2.86-4.82, n = 34; 1-hr TS: 5.81, 4.87-6.93, n = 26; 2-hr TS: 9.36, 8.27-10.59, n = 27). Values that differ significantly from WT (Kolmogorov-Smirnov test) are shown above each data point. ***p < 0.001. **(G)** Timeline illustrating the TS paradigm used for *twk-18(cn110ts)*. L4 worms were imaged before (TS) (baseline, TS = 0 hrs), after a TS to 30°C for 0.5-hr, or after a recovery (Rec) TS to 20°C for 0.5, 1, or 2 hrs. **(H)** Representative images showing SEP fluorescence (surface GLR-1) in the AVA processes in the posterior VNC in animals expressing a SEP::mCherry::GLR-1 integrated transgene (*akIs201*) in WT and *twk-18(cn110ts)* temperature sensitive mutants at baseline, after TS to 30°C (0.5 hr), and after increasing duration of Recovery at 20°C (0.5, 1, or 2 hrs). **(I)** Quantification of SEP fluorescence (surface GLR-1) at each imaging timepoint for strains shown in **H.** The graph shows Geometric mean, lower 95% CI - upper 95% CI for WT (baseline (Norm.): 1.00, 0.75-1.33, n = 28; 0.5-hr TS: 0.85, 0.50-1.44, n = 19; TS + 0.5-hr Rec: 1.39, 1.02-1.89, n = 19; TS + 1-hr Rec: 1.46, 1.11-1.91, n = 20; TS + 2-hrs Rec: 0.93, 0.67-1.30, n = 18) and *twk-18(cn110ts)* (baseline (Norm.): 1.05, 0.71-1.55, n = 26; 0.5-hr TS: 5.47, 4.31-6.94, n = 25; TS + 0.5-hr Rec: 5.16, 4.34-6.14, n = 30; TS + 1-hr Rec: 3.17, 2.59-3.89, n = 27; TS + 2-hrs Rec: 1.63, 1.24-2.15, n = 27). Values that differ significantly from WT (Kolmogorov-Smirnov test) are shown above each data point. *p < 0.05, ***p < 0.001.

First, we acutely inactivated body wall muscle using a temperature-sensitive mutant allele (*e1301ts*) of *unc-54*. *unc-54* encodes a myosin heavy chain subunit that is only expressed in body wall muscle and is required for muscle contraction [68]. Shifting *unc-54(e1301ts)* mutants to the restrictive temperature (30°C) at the L4-larval stage, when neuronal development is largely complete, results in inactivation of UNC-54 and progressively severe paralysis starting around the 2-hour timepoint. We observed that surface GLR-1 levels increase as early as 2 hours after shift to the restrictive temperature and continued to increase with longer durations of muscle inactivity (Fig 2A–2C). In contrast, surface levels (SEP fluorescence) of the NMDA receptor subunit NMR-2 were unaltered (p = 0.09) in *unc-54(e1301ts)* mutants after 4-hour shift to the restrictive temperature to inactivate muscle (S2B and S2C Fig). These data suggest that the feedback pathway can be induced with acute muscle paralysis in developed animals and that the effect may be relatively specific for GLR-1.

Second, we used a gain-of-function, temperature-sensitive mutant allele (*cn110ts*) of *twk-18* to acutely inactivate body wall muscle. TWK-18 is a hyperpolarizing potassium channel expressed in body wall muscle [69]. Shifting *twk-18(cn110ts)* mutants to the restrictive temperature (>25°C) renders the channel constitutively open, resulting in hyperpolarized muscle that is resistant to depolarization and contraction [69]. *twk-18(cn110ts)* mutants begin paralyzing within minutes of the temperature shift at the L4-larval stage and are fully paralyzed by 2 hours at the restrictive temperature. Since the *twk-18(cn110ts)* mutant results in a faster paralysis of muscle compared to the *unc-54(e1301ts)* mutant, we used *twk-18(cn110ts)* to probe the kinetics of the feedback pathway. We observed a progressive increase in surface GLR-1 levels starting as early as 0.5 hours after shift to the restrictive temperature (Fig 2D–2F). Since the temperature-induced paralysis of *twk-18* mutants is reversible – worms recover normal locomotion within 2 hours following a 0.5-hour paralysis – we tested if the effects of *twk-18* on GLR-1 surface levels were also reversible. Interestingly, using this temperature-shift paradigm we found that the increase in surface GLR-1 levels observed after 0.5-hour at the restrictive temperature gradually subsides back to baseline over a 2-hour recovery period at the permissive temperature (Fig 2G–2I). Overall, these data indicate that acute loss of muscle activity in developed animals is sufficient to trigger the feedback pathway leading to increased surface GLR-1 levels in AVA neurons. These data also indicate that the increase in surface GLR-1 can be induced on a relatively short timescale (~0.5 hour), correlates with the degree of muscle paralysis, and appears to require a continuous signal to maintain surface GLR-1 levels.

Third, we used a chemical-genetic system to inactivate muscle in the absence of any temperature shifts. Ectopic expression of the *Drosophila* Histamine-gated chloride channel (HisCl1) in *C. elegans* neurons can be used to acutely inactivate neurons upon addition of exogenous Histamine [70]. We generated transgenic animals expressing HisCl1 in muscle (using the *myo-3* promoter) and treated them with exogenous Histamine for 0.5-4 hrs starting at the L4-larval stage. We found that Histamine treatment results in a mild paralysis and increasing surface levels (SEP fluorescence) of GLR-1 starting at 1 hour (S4 Fig). Together, these data show that acute inactivation of muscle in developed animals using three independent approaches results in increased surface levels of GLR-1 in AVA neurons.

### The feedback pathway is dependent on the DCV regulator *unc-31*/CAPS

**Fig 3:**
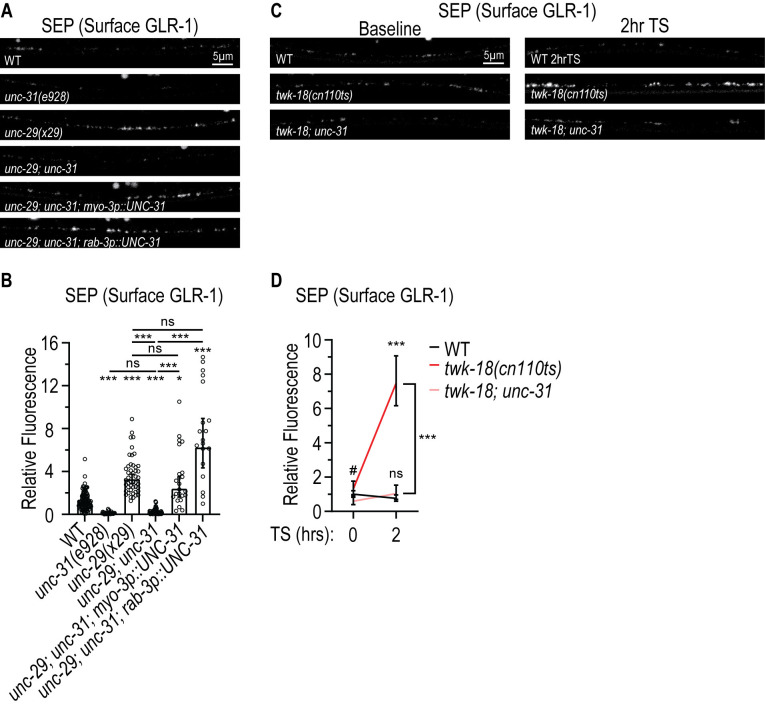
The feedback pathway triggered by loss of *unc-29*/AChRs is dependent on the DCV regulator *unc-31*/CAPS. **(A)** Representative images showing SEP fluorescence (surface GLR-1) in AVA processes in the posterior VNC in animals expressing a SEP::mCherry::GLR-1 integrated transgene (*akIs201*) in WT, *unc-31(e928)*, *unc-29(x29)*, *unc-29(x29); unc-31(e928)*, and *unc-29(x29); unc-31(e928)* mutants expressing WT *unc-31 cDNA* under control of the body wall muscle *myo-3* promoter (*pzEx479: myo-3p::UNC-31)* or under control of the pan-neuronal *rab-3* promoter (*pzEx498: rab-3p::UNC-31*). **(B)** Quantification of SEP fluorescence (Normalized) for the strains shown in **A.** The graph shows Geometric mean, lower 95% CI - upper 95% CI for WT (1.00, 0.87-1.15, n = 109), *unc-31(e928)* (0.09, 0.06-0.13, n = 28), *unc-29(x29)* (3.27, 2.86-3.74, n = 47), *unc-29(x29); unc-31(e928)* (0.16, 0.13-0.20, n = 74), and *unc-29(x29); unc-31(e928)* animals expressing *unc-31* cDNA under control of the *myo-3* promoter (*pzEx479: myo-3p::UNC-31)*(2.39, 1.62-3.52, n = 23) or under control of the *rab-3* promoter (*pzEx498: rab-3p::UNC-31*)(6.23, 4.34-8.94, n = 19). Values that differ significantly from WT (Kruskal-Wallis, Dunn’s multiple comparison test) are indicated above each bar. Other comparisons are marked by brackets. ***p < 0.001, *p < 0.05, n.s., p > 0.05. **(C)** Representative images showing SEP fluorescence (surface GLR-1) in the AVA processes in the posterior VNC in animals expressing a SEP::mCherry::GLR-1 integrated transgene (*akIs201*) in WT, *twk-18(cn110ts)* temperature-sensitive mutants and *twk-18(cn110ts); unc-31(e928)* mutant animals. The worms were imaged at baseline or after a 2-hrs TS. **(D)** Quantification of SEP fluorescence (Normalized) for the strains shown in **C.** The graph shows Geometric mean, lower 95% CI - upper 95% CI for WT (baseline (Norm.): 1.00, 0.83-1.20, n = 34; 2 hrs TS: 0.76, 0.60-0.97, n = 28), *twk-18(cn110ts)* (baseline: 1.31, 0.98-1.77, n = 33; 2 hrs TS: 7.47, 6.16-9.07, n = 36), and *twk-18(cn110ts); unc-31(e928)* (baseline: 0.60, 0.40-0.90, n = 19; 2 hrs TS: 1.04, 0.70-1.53, n = 28) animals at baseline and after a 2-hrs TS. Values that differ significantly from WT (data log-transformed to perform Two-way ANOVA with Sidak’s multiple comparison test) are shown above each data point. Other comparisons are indicated by a bracket. ***p < 0.001, n.s. p > 0.05. # indicates statistical significance (p < 0.01) between *twk-18(cn110ts)* and *twk-18(cn110ts); unc-31* at baseline.

Because AVA resides two synaptic layers upstream of the NMJ, we hypothesized that the feedback pathway is mediated by a factor secreted from muscle that can reach AVA neurons to promote surface GLR-1 levels. Several previous studies in *C. elegans* have implicated neuropeptides in inter-tissue signaling between the intestine [21–26] or body wall muscle [42,49,71] and the nervous system. Neuropeptides are packaged into dense-core vesicles (DCVs) and undergo a complex biogenesis and maturation process prior to being trafficked and released at extrasynaptic sites [72,73]. The secretion of most, but not all, neuropeptide-containing DCVs in worms [74], flies [75] and mammals [76] is mediated by Calcium Activator for Protein Secretion (CAPS). To test whether neuropeptides mediate the feedback pathway, we analyzed surface levels of GLR-1 in AVA in the absence of *unc-31,* the *C. elegans* homolog of CAPS, after triggering the feedback pathway (i.e., in *unc-29*/AChR mutants). We found that the increase in SEP fluorescence (surface GLR-1 levels) observed in *unc-29*/AChR mutants was completely blocked by *unc-31*/CAPS mutation (90% decrease) (Fig 3A and 3B, 3^rd^ vs 4^th^ bars). These data suggest that *unc-31*/CAPS and the feedback mechanism triggered in *unc-29*/AChR mutants act in the same genetic pathway and that the increase in surface GLR-1 triggered by loss of NMJ signaling is dependent on DCV release. Intriguingly, expression of wild-type *unc-31* cDNA specifically in body wall muscle (using the muscle specific promoter, *myo-3p*) or neurons (using the pan-neuronal promoter *rab-3p*) was sufficient to rescue the decrease in surface GLR-1 in *unc-29;unc-31* double mutants (Fig 3A and 3B). Additionally, we found that surface GLR-1 levels were decreased in *unc-31*/CAPS single mutants (~90% decrease) (Figs 3A–3B and S5). However, this decrease in surface GLR-1 was rescued by expression of wild-type *unc-31* cDNA in neurons but not in muscle (S5 Fig). These data suggest that *unc-31*/CAPS can act in neurons to release one or more neuropeptides that promote surface GLR-1 levels basally but that *unc-31*/CAPS acts in both neurons and muscle to promote surface GLR-1 when the feedback pathway is triggered. Although multiple neuropeptides are likely released in an *unc-31/*CAPS-dependent manner from both neurons and muscle to regulate surface GLR-1 levels, the ability of muscle-expressed *unc-31* to rescue surface GLR-1 levels in *unc-31* mutants only occurs when the feedback pathway is triggered (i.e., in *unc-29*/AChR mutants)(Figs 3A and 3B and S5), perhaps because the trigger increases expression or function of UNC-31/CAPS or its relevant secreted cargo in muscle.

We next tested whether the feedback pathway induced by acute loss of muscle function was also mediated by *unc-31*/CAPS. We found that the increase in surface GLR-1 observed in *twk-18(cn110ts)* mutants after a paralyzing 2-hour shift to the restrictive temperature is also blocked in *unc-31*/CAPS mutants (Fig 3C and 3D). Together, these results are consistent with the idea that *unc-31*/CAPS-dependent DCV secretion of neuropeptides or other factors from neurons or body wall muscle are required to mediate the feedback pathway.

Because we previously found that PVF-1/VEGF is released from body wall muscle to promote surface GLR-1 levels in AVA neurons [49], we tested if PVF-1 is the muscle-secreted factor that mediates the feedback pathway. Unexpectedly, we found that loss of *pvf-1* does not suppress the increased surface GLR-1 levels in the VNC triggered by *unc-29(x29)*/AChR mutants (Geometric mean SEP fluorescence, lower 95% CI – upper 95% CI (Norm.)): WT: 1.0, 0.8-1.3; *unc-29(x29):* 3.6, 2.5-5.2, p < 0.001 vs WT; *unc-29(x29);pvf-1(ev763)*: 3.0, 2.0-4.4; p = 0.001 vs WT, ns p > 0.05 vs *unc-29*). This result indicates that *pvf-1* is not required to mediate the feedback pathway and suggests the involvement of another muscle-secreted factor dependent on *unc-31*/CAPS, such as a neuropeptide.

### Loss of the muscle-expressed insulin-like peptide *ins-27* results in decreased surface GLR-1 in AVA and defects in glutamatergic behavior

**Fig 4:**
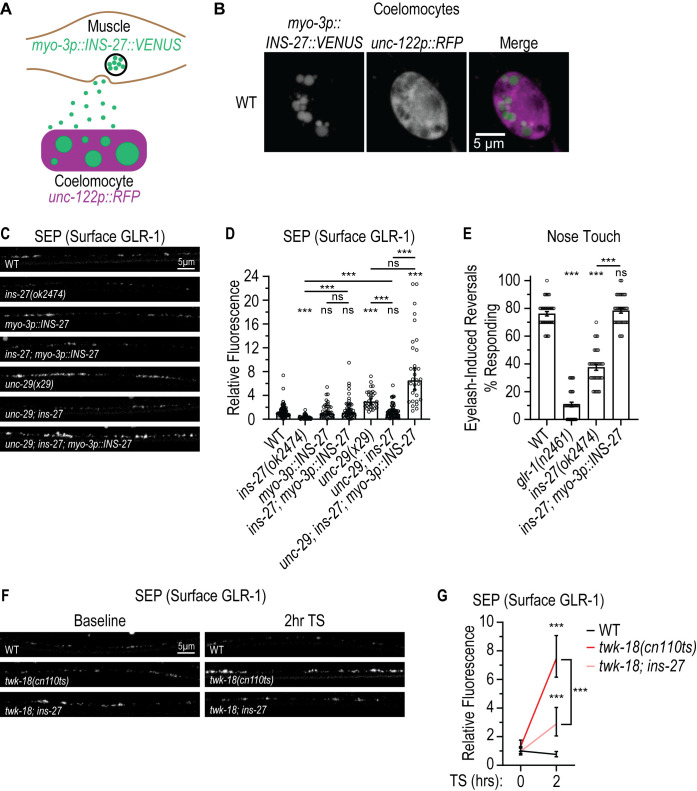
Loss of the muscle-expressed insulin-like peptide *ins-27* results in defects in glutamatergic behavior and suppresses the feedback pathway. (**A)** Depiction of the coelomocyte assay for secreted peptides. INS-27::VENUS is expressed specifically in body wall muscles using the *myo-3* promoter (*myo-3p::INS-27::VENUS*). RFP marks the coelomocytes (*unc-122p::RFP*). INS-27::VENUS secreted from muscle into the pseudocoelom is taken up by coelomocytes. The amount of INS-27::VENUS secreted from muscle can be estimated by measuring the accumulation of INS-27::VENUS in vesicles within coelomocytes. **(B)** Representative images showing that INS-27::VENUS is released from the muscle and accumulates in coelomocytes in WT animals expressing INS-27::VENUS in body wall muscle (*pzEx488: myo-3p::INS-27::VENUS*). Images show INS-27::VENUS fluorescence (left panel), RFP fluorescence marking coelomocytes (middle panel) and a merged image of both channels (right panel). **(C)** Representative images showing SEP fluorescence (surface GLR-1) in the AVA processes in the posterior VNC in animals expressing a SEP::mCherry::GLR-1 integrated transgene (*akIs201*) in WT, *ins-27(ok2474)*, WT animals expressing *myo-3p::INS-27*, *ins-27(ok2474)* mutant animals expressing *myo-3p::INS-27, unc-29(x29)*, *unc-29(x29); ins-27(ok2474)*, and *unc-29(x29); ins-27(ok2474)* animals expressing *myo-3p::INS-27*. **(D)** Quantification of SEP fluorescence (Surface GLR-1) for strains shown in **C.** The graph shows Geometric mean, lower 95% CI - upper 95% CI for WT (1.00, 0.86-1.16, n = 96), *ins-27(ok2474)* (0.20, 0.15-0.27, n = 54), *pzEx484 (myo-3p::INS-27)* (0.98, 0.71-1.37, n = 40), *ins-27(ok2474); pzEx484 (myo-3p::INS-27)*(1.12, 0.81-1.55, n = 49), *unc-29(x29)* (2.99, 2.53-3.54, n = 31), *unc-29(x29); ins-27(ok2474)* (0.93, 0.75-1.16, n = 65), and *unc-29(x29); ins-27(ok2474); pzEx484 (myo-3p::INS-27)* (6.52, 4.94-8.61, n = 31). Values that differ significantly from WT (Kruskal-Wallis, Dunn’s multiple comparison test) are indicated above each bar. Other comparisons are marked by brackets. n.s. p > 0.05, ***p < 0.001. **(E)** Quantification of eyelash-induced reversals in the nose touch assay. Each data point corresponds to the percentage (%) of reversals after 10 trials. WT: 76.11 ± 1.61; *glr-1(n2461)*: 10.83 ± 1.66; *ins-27(ok2474)*: 37.50 ± 2.12; *ins-27(ok2474); pzEx484 (myo-3p::INS-27):* 78.33 ± 1.76. n = 36 for all. The graph shows Mean Percentage ± SEM. Values that differ significantly from WT (One-way ANOVA, Sidak’s multiple comparison test) are indicated above each bar. Other comparisons are marked by brackets. ***p < 0.001. **(F)** Representative images showing SEP fluorescence (surface GLR-1) in the AVA processes in the posterior VNC in animals expressing a SEP::mCherry::GLR-1 integrated transgene (*akIs201*) in WT, *twk-18(cn110ts)* temperature-sensitive mutants and *twk-18(cn110ts); ins-27(ok2474)* mutant animals. The worms were imaged at baseline or after a 2-hrs TS. **(G)** Quantification of SEP fluorescence (Surface GLR-1) for strains shown in **F.** The graph shows Geometric mean, lower 95% CI - upper 95% CI for WT (baseline (Norm.): 1.00, 0.83-1.20, n = 34; 2-hrs TS: 0.76, 0.60-0.97, n = 28), *twk-18(cn110 ts)* (baseline: 1.31, 0.98-1.77, n = 33; 2-hrs TS: 7.47, 6.16-9.07, n = 36), and *twk-18(cn110ts); ins-27(ok2474)* (baseline: 0.97, 0.73-1.30, n = 26; 2-hrs TS: 2.88, 2.06-4.04, n = 30) worms at baseline and after a 2-hrs TS. Values that differ significantly from WT (data log-transformed to perform Two-way ANOVA with Sidak’s multiple comparison test) are shown above each data point. Other comparisons are marked by a bracket. ***p < 0.001.

The *C. elegans* genome encodes over 100 neuropeptide genes that result in more than 200 neuropeptides [77–79]. These neuropeptides are categorized into three peptide families: the insulin-like peptide *ins*, neuropeptide-like peptide *nlp*, and FMRFamide-like peptide *flp* families [72,79]. At least 40 of these neuropeptides are expressed in body wall muscle [80], however the functions of these muscle-derived peptides have not been characterized. Using a candidate gene approach, we identified the insulin-like peptide *ins-27* as a potential mediator of the feedback pathway. *ins*-27 is one of the most highly expressed neuropeptides in body wall muscle [80,81]. We first tested if INS-27 protein could be secreted from body wall muscle. Secretion of proteins in *C. elegans* can be measured with an established assay where accumulation of the secreted protein is measured in coelomocytes [82](Fig 4A). Coelomocytes are specialized scavenger cells that endocytose fluid from the body cavity (pseudocoelom). Changes in protein secretion from neurons or other cells can be indirectly monitored by measuring the accumulation of fluorescently tagged-neuropeptides that have been internalized by coelomocytes. For example, mutants that increase or decrease DCV secretion of neuropeptides result in corresponding changes in neuropeptide fluorescence in coelomocytes [82,83]. We expressed Venus-tagged INS-27 specifically in body wall muscle (using the *myo-3* promoter) and tested whether INS-27::Venus would accumulate in coelomocytes (marked with *unc-122p::RFP*)(Fig 4A). We were able to detect INS-27::VENUS accumulation in coelomocytes (Fig 4B), indicating that INS-27 is secreted from body wall muscle and taken up by coelomocytes.

We next tested whether loss of *ins-27* alone had an effect on surface levels of GLR-1 using a loss-of-function allele of *ins-27(ok2474)*. *ok2474* is comprised of a 461 bp deletion that removes the entire coding sequence of *ins-*27 and thus represents a null allele. The deletion starts 146 bp upstream of the start codon and ends in the 3’UTR of the *ins-27* locus. We found that *ins-27(ok2474)* mutants exhibit decreased surface GLR-1 levels (~80% decrease) in AVA neurons and this defect can be rescued by expression of wild-type *ins-27* cDNA specifically in body wall muscle (using the *myo-3* promoter)(Fig 4C and 4D, 2^nd^ and 4^th^ bars). We investigated whether this decrease in surface GLR-1 altered glutamatergic behavior using the nose-touch response. Activation of the glutamatergic sensory neuron ASH with mechanical stimuli (i.e., using an eyelash) results in activation of the pre-motor interneuron AVA and backward locomotion away from the stimulus [51,52,54,55,58]. Worms that are defective in glutamate signaling, such as mutants lacking presynaptic glutamate release (i.e., *eat-4*/VGLUT) [55] or postsynaptic glutamate receptors (i.e., *glr-1*/GluRs)(Fig 4E) [51,52], or mutants that have decreased surface GLR-1 at synapses [49,84,85] exhibit defects in the nose-touch response. We found that *ins-27* loss-of-function mutants exhibit defects in the nose-touch response and this defect could be rescued by expression of wild-type *ins-27* cDNA in body wall muscle (Fig 4E). Collectively, these results indicate that loss of one of the most highly expressed neuropeptides in muscle, *ins-27*, results in a decrease in surface GLR-1 levels in AVA neurons that is correlated with defects in glutamatergic nose-touch behavior.

### The feedback pathway is dependent on *ins-27* and the *daf-2*/Insulin/IGF-1 receptor

Next, we tested whether INS-27 mediates the feedback pathway. We found that loss of *ins-27* partially suppresses the increase in surface GLR-1 levels triggered by loss of NMJ signaling in *unc-29*/AChR mutants (~70% decrease)(Fig 4C and 4D, compare 5^th^ vs 6^th^ bars). This suppression is relieved when wild-type *ins-27* is expressed specifically in body wall muscle (Fig 4C and 4D, compare 6^th^ vs 7^th^ bars), suggesting that INS-27 is required for the feedback pathway and can potentially signal from the muscle to regulate surface GLR-1 levels in AVA neurons. Interestingly, overexpression of *ins-27* in the body wall muscle of wild-type animals is not sufficient to increase surface GLR-1 levels above wild-type levels (Fig 4D, 3^rd^ bar), but overexpression of *ins-27* in body wall muscle in *ins-27* mutants can increase surface GLR-1 levels above the wild-type level when the feedback pathway is triggered (i.e., in *unc-29*/AChR mutants)(Fig 4D, 7^th^ bar). This data is consistent with the idea that INS-27 secretion or function can somehow be increased when the feedback pathway is triggered.

We also investigated whether loss of *ins-27* suppresses the increased surface GLR-1 levels triggered by acute muscle inactivation in *twk-18* temperature-sensitive gain-of-function potassium channel mutants. We found that loss of *ins-27* also partially suppresses the increase in surface GLR-1 levels in AVA triggered by *twk-18* mutants at the restrictive temperature (Fig 4F and 4G). Together, these results are consistent with INS-27 being a neuropeptide that is released from muscle that mediates the feedback pathway to promote surface levels of GLR-1 in AVA neurons.

**Fig 5:**
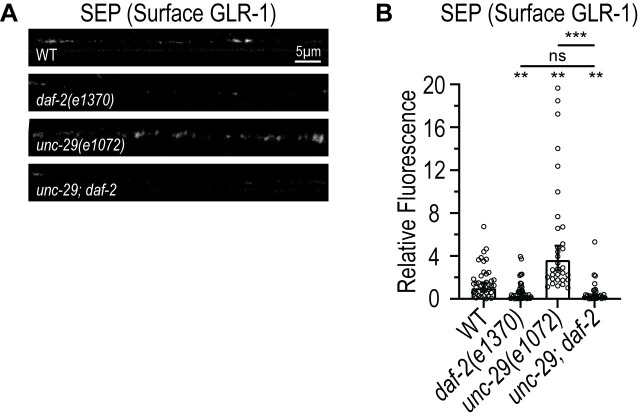
The feedback pathway triggered by loss of *unc-29*/AChRs is dependent on the *daf-2*/Insulin/IGF-1 receptor. **(A)** Representative images showing SEP fluorescence (surface GLR-1) in the AVA processes in the posterior VNC in animals expressing a SEP::mCherry::GLR-1 integrated transgene (*akIs201*) in WT, *daf-2(e1370)*, *unc-29(e1072)* and *unc-29(e1072); daf-2(e1370)* animals. **(B)** Quantification of SEP fluorescence (Surface GLR-1) for strains shown in **A.** The graph shows Geometric mean, lower 95% CI - upper 95% CI for WT (1.00, 0.66-1.52, n = 38), *daf-2(e1370)* (0.26, 0.15-0.46, n = 37), *unc-29(e1072)* (3.62, 2.65-4.96, n = 32), and *unc-29(e1072);daf-2(e1370)* (0.23, 0.12-0.42, n = 29). Values that differ significantly from WT (Kruskal-Wallis test, Dunn’s multiple comparison test) are indicated above each bar. Other comparisons are marked by brackets. n.s. p > 0.05, **p < 0.01, ***p < 0.001.

In *C. elegans*, all the ILP’s including INS-27 are thought to act via DAF-2, which is the sole Insulin/IGF-1 receptor in the worm [86,87]. DAF-2 is widely expressed in *C. elegans* including in AVA and other neurons. We hypothesized that if the effects of INS-27 are mediated by DAF-2, then loss of *daf-2* would be expected to block the feedback pathway. We found that *daf-2(e1370)* loss-of-function mutants exhibit decreased surface GLR-1 levels and block the increase in surface GLR-1 triggered in *unc-29*/AChR mutants (Fig 5A and 5B). The *unc-29;daf-2* double mutants are indistinguishable from *daf-2* single mutants (ns, p > 0.05) suggesting that *daf-2* is required for the ability of the feedback pathway to increase surface GLR-1 levels in AVA neurons. Together, these data are consistent with a model where INS-27 acts via DAF-2 receptors to mediate the feedback pathway. Interestingly, our data showing that *ins-27* mutants partially block (Fig 4C, 4D, 4F and 4G) whereas *daf-2* mutants completely block (Fig 5A and 5B) the feedback pathway suggest that INS-27 acts together with another ILP to mediate the feedback pathway through DAF-2 receptors.

### INS-27 secretion from muscle is stimulated by chronic loss of NMJ signaling or acute inactivation of muscle and is dependent on the DCV regulator *unc-31*/CAPS

**Fig 6:**
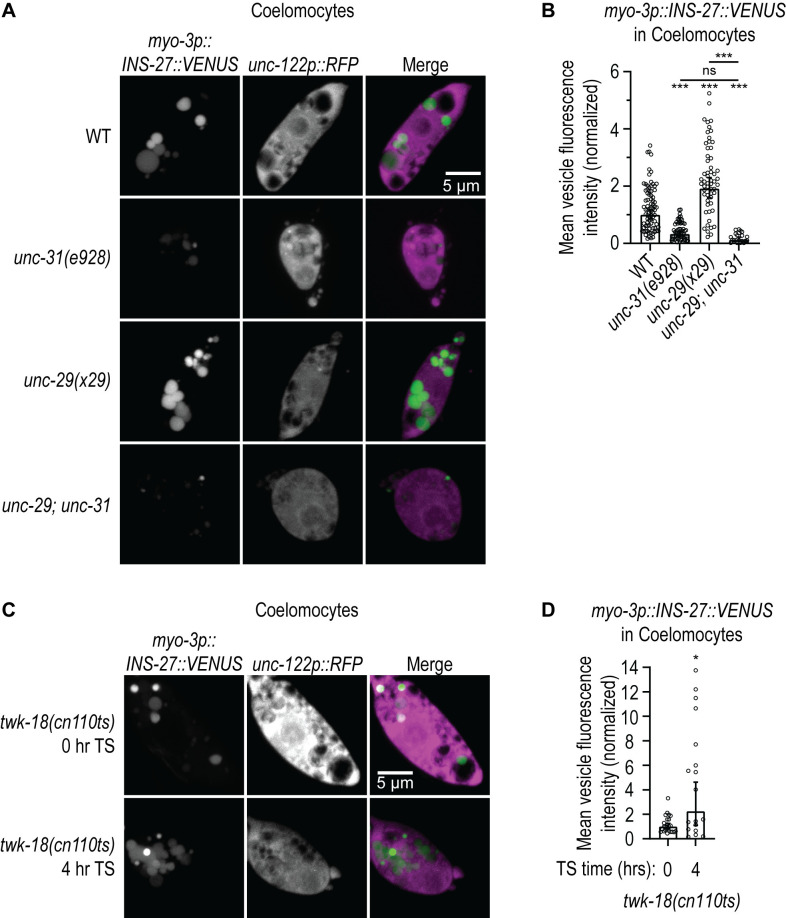
INS-27 secretion from muscle is dependent on *unc-31*/CAPS and stimulated by loss of NMJ signaling or acute loss of muscle activity. **(A)** Representative images showing INS-27::VENUS accumulation in coelomocytes (marked by *unc-122p::RFP*) in animals expressing INS-27::VENUS in body wall muscle (*pzEx488: myo-3p::INS-27::VENUS*) in WT, *unc-31(e928)*, *unc-29(x29)* and *unc-29(x29); unc-31(e928)* mutant animals. Images show INS-27::VENUS fluorescence (left panels), coelomocytes marked by RFP fluorescence (middle panels) and a merged image of both channels (right panels). **(B)** Quantification of the mean INS-27::VENUS fluorescence intensity in coelomocytes in strains shown in **A.** The graph shows Geometric mean, lower 95% CI - upper 95% CI for WT (1.00, 0.85-1.17, n = 82), *unc-31(e928)* (0.33, 0.26-0.40, n = 58), *unc-29(x29)* (1.91, 1.59-2.30, n = 57), and *unc-29(x29); unc-31(e928)* (0.13, 0.07-0.22, n = 21). Values that differ significantly from WT (Kruskal-Wallis, Dunn’s multiple comparison test) are shown above each data point. Other comparisons are marked by a bracket. ***p < 0.001, n.s., p > 0.05. **(C)** Representative images showing INS-27::VENUS accumulation in coelomocytes (marked by *unc-122p::RFP*) in animals expressing INS-27::VENUS in body wall muscle (*pzEx488: myo-3p::INS-27::VENUS*) in *twk-18(cn110ts)* mutant animals before (0hr) and after (4hr) shift to the restrictive temperature (TS). Images show INS-27::VENUS fluorescence (left panels), coelomocytes marked by RFP fluorescence (middle panels) and a merged image of both channels (right panels). **(D)** Quantification of the mean INS-27::VENUS fluorescence intensity in coelomocytes in strains shown in **C.** The graph shows Geometric mean, lower 95% CI - upper 95% CI for *twk-18(cn110ts)* 0h (1.00, 0.80-1.26, n = 24) and *twk-18(cn110ts)* 4h TS (2.25, 1.09-4.63, n = 18). Values that differ significantly from control (Kolmogorov-Smirnov test) are shown above each data point. *p < 0.05.

Our data suggest that *unc-*31/CAPS can act in muscle to mediate the feedback pathway. We tested if secretion of INS-27::VENUS from muscle (*myo-3p::INS-27::Venus*) is dependent on *unc-31*/CAPS using the coelomocyte assay described above. We found that loss of *unc-31*/CAPS leads to a reduction in the amount of INS-27::VENUS in coelomocytes (Fig 6A and 6B), suggesting that *unc-31*/CAPS-dependent DCV release is required for the secretion of INS-27::VENUS from muscle.

If INS-27 mediates the muscle-to-neuron feedback pathway in an instructive manner then we would expect that loss of NMJ signaling may stimulate the secretion of INS-27 from muscle. Indeed, we found that loss of NMJ signaling in *unc-29*/AChR mutants results in an increase in INS-27::VENUS fluorescence in coelomocytes and this increase was blocked by loss of *unc-31*/CAPS (Fig 6A and 6B). These data suggest that loss of NMJ signaling triggers increased secretion of INS-27::VENUS from muscle in a process that requires *unc-31*-dependent DCV release. We also tested if acute inactivation of muscle in developed animals using the temperature-sensitive gain-of-function *twk-18* potassium channel mutant could promote release of INS-27 from muscle. We found that INS-27::VENUS fluorescence in coelomocytes increases after shifting *twk-18(cn110ts)* mutants to the restrictive temperature for 4 hours (Fig 6C and 6D) suggesting that acute inactivation of muscle in developed animals can also trigger an increase in secretion of INS-27 from muscle. Collectively, our results reveal a potential muscle-to-neuron feedback signal and support a model where loss of NMJ signaling or muscle activity leads to the *unc-31*/CAPS-dependent release of INS-27 from muscle that acts via DAF-2 insulin/IGF-1 receptors to promote surface levels of GLR-1 in upstream AVA pre-motor interneurons.

## Discussion

Recent evidence shows that AMPAR trafficking and clustering at synapses can be regulated extrinsically by a number of secreted factors. Although most of these factors are secreted from neurons or glia that act in an autocrine or paracrine manner on nearby synapses [4,18], it is less clear whether these factors also have effects on distal neurons or if AMPARs can be regulated by factors secreted from other non-neuronal cells and tissues. In this study, we discovered an extracellular signal in *C. elegans* that is secreted from muscle that regulates surface levels of AMPARs in a pair of pre-motor interneurons, AVA, that reside two synaptic layers upstream of the NMJ. We found that loss of NMJ signaling (Fig 1) or muscle activity (Figs 2 and S4) triggers a muscle-to-neuron signal that results in increased surface GLR-1 levels in AVA neurons. This increase in surface levels of GLR-1 is unlikely to be a secondary consequence of a defect in development because it can be triggered by acute loss of muscle activity (i.e., using a conditional loss-of-function *unc-54*/myosin mutant, a conditional gain-of-function *twk-18*/potassium channel mutant or chemical-genetic inhibition of muscle activity)(Figs 2 and S4) after nervous system development is largely complete. The increase in surface GLR-1 can be triggered relatively quickly (within 30 minutes) and is reversible, suggesting that the pathway needs to be continuously triggered to promote or maintain increased surface GLR-1 levels. It remains an open question how signaling is rapidly terminated after inactivation of the muscle trigger.

Our findings are consistent with a model where loss of muscle activity results in activation of a signaling pathway that increases levels of GLR-1 at the postsynaptic membrane of AVA, perhaps as a compensatory feedback mechanism to increase the drive on the motor circuit. In *C. elegans*, several other feedback pathways have been described including a mechanosensory stretch-activated pathway in vulva muscles that coordinates development and activity of the egg laying circuit [88,89], a stretch responsive neuron that couples muscle contraction to presynaptic potentiation mediated by the neuropeptide NLP-12 [90], and a sensorimotor feedback loop that coordinates forward locomotion that is mediated by a proprioceptive signal from a subset of cholinergic motor neurons [91]. In our study, we found that loss of muscle activity results in increased surface levels of GLR-1 at synapses distributed globally throughout AVA neurites (Fig 1). We speculate that this global increase in GLR-1 may enhance the sensitivity of AVA to all upstream glutamatergic sensory neuron inputs as a mechanism to promote locomotion in the face of decreased muscle function. This global compensatory increase in GLR-1 is reminiscent of synaptic scaling where chronic decreases in synaptic activity leads to global scaling up of all synapses in a neuron via increased AMPAR surface levels [92,93]. However, in our study the increase in AMPAR surface levels occurs throughout AVA neurons that reside two synaptic layers upstream of the muscle cells experiencing the chronic decrease in activity, providing an example of a compensatory signal that acts on distal neurons in a circuit beyond the most immediately-connected cells.

### The muscle-to-neuron feedback signal is dependent on the DCV regulator UNC-31/CAPS

Our data show that loss of *unc-31*/CAPS blocks the feedback pathway to increase surface GLR-1 levels in AVA neurons and this effect can be reversed by expression of wild-type *unc-31* in neurons or body wall muscle (Fig 3). Considering that UNC-31/CAPS mediates DCV release in *C. elegans*, *Drosophila,* and mammals [74–76], our data suggest that *unc-31* can function in neurons and muscle to regulate the release of neuropeptides that promote surface GLR-1 levels. It would not be too surprising if several neuropeptides are involved in regulating GLR-1 given that multiple neuropeptides are known to regulate synaptic transmission at other synapses such as the NMJ [94–96]. Although our study focused on identifying neuropeptides released from muscle that mediate the feedback pathway, it will be interesting in the future to identify the neuropeptides released from neurons that also regulate GLR-1. Given that the stretch sensitive neuron DVA releases the neuropeptide NLP-12 to regulate cholinergic transmission in response to muscle contraction [90], it is possible that proprioceptive neurons may detect changes in muscle contraction and release neuropeptides that also contribute to the feedback pathway regulating GLR-1.

Retrograde signaling between postsynaptic and presynaptic cells coordinates synapse development and function at neuron-neuron synapses and at the NMJ in vertebrates and invertebrates [97,98]. Retrograde signals include nitric oxide [99], endocannabinoids [100], neurotransmitters [101], and DCV-dependent protein factors [102–104]. Several studies have implicated DCVs and neuropeptides in signaling from muscle. For example, *C. elegans* muscle can regulate presynaptic function at the NMJ via a retrograde neuropeptide signal that requires the neuropeptide processing enzyme *aex-5*/pro-protein convertase and the calcium-binding DCV regulator *aex-1*/Munc13b [41,42]; however, the identity of the neuropeptide that mediates this retrograde signal is not yet known. Interestingly, studies at the fly NMJ showed that synaptotagmin IV [97] and the SNARE syntaxin 4 [105] act in muscle to mediate a retrograde signal to motor neurons to regulate synapse growth and plasticity. This retrograde signal could be mediated by DCVs as mammalian synaptotagmin IV has been shown to regulate DCV release from hippocampal neurons [106] and pituitary cells [107,108]. Other studies in *C. elegans* revealed the existence of a long-range signal that couples body wall muscle and the pharynx, a muscular feeding organ in the head of the animal. Changes in cholinergic signaling at the NMJ [109] or optical silencing of body wall muscle [71] triggers a signal that inhibits pumping of the pharynx, which may serve to inhibit feeding during locomotion. Interestingly, Takahashi et al. (2017) found that this long-range signal is partially dependent on *unc-31*/CAPS [71], suggesting that the signal is mediated by a factor secreted from DCVs. Although Izquierdo et al. (2022) were not able to identify the secreted factor that mediates the NMJ-to-pharynx signal, they were able to rule out several classes of secreted factors including classical neurotransmitters, biogenic amines (i.e., dopamine, serotonin or tyramine) and the subset of neuropeptides that are processed by the pro-protein convertase *egl-3*/PPC [109]. Collectively, these studies are consistent with roles for DCVs and neuropeptides signaling from muscle, however the identity of the specific neuropeptides that are released from muscle to mediate these various signals have remained elusive.

### The muscle-to-neuron feedback signal is dependent on the insulin-like peptide INS-27 and the insulin/IGF-1 receptor DAF-2

Using a candidate approach, we identified the ILP INS-27 as a potential mediator of the feedback pathway. In *C. elegans*, ILPs regulate several processes including development, neuronal function, aging and metabolism [110]. *C. elegans* express 40 ILPs, however the biological function of most of these peptides are incompletely understood [110,111]. A few large-scale studies of the ILP family have investigated potential functions for INS-27. One prior study overexpressed each of the 40 ILPs in the nervous system of *C. elegans* and found that pan-neuronal overexpression of *ins-27* does not result in any observable phenotypes related to canonical insulin signaling (i.e., dauer formation, longevity, and fat storage) [87]. Another large-scale survey of the ILP’s using loss-of-function mutants implicated INS-27 in thermotolerance and pathogen resistance [112].

Our data reveal a novel function for INS-27. Several lines of evidence support a model where INS-27 mediates a feedback signal between muscle and upstream neurons by promoting surface levels of GLR-1/AMPARs in AVA neurons. First, INS-27 is the most highly expressed neuropeptide in muscle [80,81] and can be secreted from muscle (Fig 4B). Second, *ins-27* loss-of-function mutants exhibit decreased surface levels of GLR-1 in AVA neurons (Fig 4C and 4D). Third, *ins-27* loss-of-function mutants exhibit defects in the nose-touch behavior (Fig 4E), which is dependent on endogenous GLR-1 function. Fourth, the defects in surface GLR-1 and GLR-1-dependent behavior observed in *ins-27* mutants can be rescued by expression of *ins-27* in body wall muscle (Fig 4C–4E). Fifth, *ins-27* mutants block the increased surface GLR-1 levels triggered by *unc-29*/AChR mutants and this suppression can be alleviated by expression of *ins-27* in body wall muscle (Fig 4C and 4D). Sixth, INS-27 secretion from muscle depends on *unc-31*/CAPS and can be triggered by loss of NMJ signaling or loss of muscle activity (Fig 6). Given that INS-27 is a secreted protein, we acknowledge that our rescue data do not rule out the possibility that INS-27 is secreted from other cells in addition to muscle. Nevertheless, our data showing that INS-27 secretion from muscle is dependent *unc-31*/CAPS and that secretion is stimulated by loss of NMJ signaling or loss of muscle activity (Fig 6) support a model where INS-27 is secreted from muscle to mediate the feedback signal. In addition, we show that *unc-31*/CAPS mutants block the increase in surface GLR-1 triggered by loss of *unc-29*/AChR signaling and that this block can be relieved by expression of *unc-31* in muscle (Fig 3). This suggests that *unc-31*/CAPS can act in muscle to promote INS-27 release and surface levels of GLR-1 when the feedback pathway is triggered.

Interestingly, loss of *ins-27* results in decreased surface GLR-1 levels under basal conditions and when the feedback pathway is triggered by loss of NMJ signaling. The fact that the decrease in surface GLR-1 levels in *ins-27* single mutants vs wild-type (~80% decrease), and *unc-29;ins-27* double mutants vs *unc-29* single mutants (~70% decrease)(Fig 4D) are similar, could be interpreted to mean that *ins-27* regulates basal levels of GLR-1 independent of the feedback pathway. However, while INS-27 may have more than one function, our data show that loss of NMJ signaling or loss of muscle activity results in increased secretion of INS-27 from muscle in an *unc-31*/CAPS-dependent manner (Fig 6). Furthermore, we found that overexpression of *ins-27* in muscle in wild-type animals is not sufficient to promote surface levels of GLR-1 above wild-type levels (Fig 4), whereas muscle-expression of *ins-27* can increase surface GLR-1 levels above a wild-type set point when the feedback pathway is triggered by *unc-29*/AChR mutants (Fig 4D). Together, these data support a more direct connection between the feedback trigger and INS-27.

One limitation of our study is that prior single cell expression analyses suggest that *unc-31* expression is relatively low in body wall muscle [80]. Interestingly, low levels of *unc-31* expression may be sufficient to regulate DCV release from some tissues as a recent study showed that UNC-31/CAPS can act in the epidermis to regulate secretion of neuropeptides even though single cell expression data indicate that the level of *unc-31* expression in the epidermis is low [113]. Nevertheless, it remains possible that endogenous *unc-31*/CAPS may not act in muscle but instead may act in another tissue, such as neurons, to indirectly regulate DCV release from muscle. In this scenario, because INS-27 secretion from muscle is blocked in *unc-31* mutants (Fig 6), neuronal UNC-31/CAPS would have to regulate the release of a factor from neurons that subsequently acted on muscle to regulate the release of INS-27. It is also possible, in this scenario, that overexpression of *unc-*31 in muscle allows UNC-31 to substitute for another endogenous DCV release factor and promote secretion of INS-27. Alternatively, UNC-31/CAPS function in muscle may be upregulated under certain conditions. Consistent with this latter possibility, our data show that muscle-expressed *unc-31* is only able to rescue surface GLR-1 levels in *unc-31* mutants when the feedback pathway is triggered (i.e., in *unc-29*/AChR mutants)(Figs 3B and S5), suggesting that the feedback pathway somehow promotes UNC-31 function in muscle.

Interestingly, there may be another secreted factor that acts together with INS-27 to mediate the feedback signal. Our data show that *unc-31*/CAPS mutants completely block the increase in surface GLR-1 triggered by loss of NMJ signaling (Fig 3B, compare 2^nd^ and 4^th^ bars, p > 0.05), whereas *ins-27* mutants only partially block (Fig 4D, compare 2^nd^ and 6^th^ bars, p < 0.001). These data hint that there may be other UNC-31-dependent secreted factors that act with INS-27 to mediate the feedback signal and these factors could be secreted from neurons or muscle.

Insulin and insulin-like growth factors can regulate learning and memory in *C. elegans* [28,114–119], *Drosophila* [120] and mammals [121,122]. In *C. elegans*, although most ILPs signal between specific neurons to mediate learning [114,115,118,119], INS-11 was shown to be released from the intestine to signal to the nervous system to regulate aversive learning behavior [28]. Our study reveals an inter-tissue signal between muscle and the nervous system. Our data are consistent with a model where loss of muscle activity triggers release of INS-27 from muscle resulting in increased surface GLR-1 levels in upstream AVA neurons. Intriguingly, our rescue data show that the ability of muscle-expressed *ins-27* to increase surface GLR-1 levels in *ins-27* mutants is enhanced in the absence of NMJ signaling (Fig 4D). This finding is consistent with the idea that triggering the feedback pathway by reducing NMJ signaling enhances the ability of INS-27 to promote surface GLR-1, perhaps via increased expression or function of UNC-31/CAPS that promotes INS-27 secretion from muscle. In support of this idea, our data show that INS-27 secretion from muscle is increased when the feedback pathway is triggered by loss of NMJ signaling or loss of muscle activity (Fig 6). In addition, the feedback pathway may increase INS-27 signaling by promoting INS-27 expression, processing, or maturation. In cultured rodent neurons, insulin has been shown to regulate AMPAR trafficking by promoting endocytosis [123]. Although INS-27 does not appear to act like a traditional insulin in *C. elegans* [87], it is not known if one or more of the other ILPs that share functions with mammalian insulin, may also regulate GLR-1 endocytosis. Regardless, although the mechanism by which INS-27 promotes GLR-1 surface levels is not known, we speculate that INS-27 may either inhibit endocytosis or promote insertion or recycling of GLR-1 to the postsynaptic membrane. It will be interesting to test if any of the other 10 mammalian insulin/insulin-like peptides [110] also promote surface levels of AMPARs. Finally, all 40 ILP’s in the worm are thought to act via DAF-2, the sole insulin receptor homolog in *C. elegans*, and DAF-2 is widely expressed throughout *C. elegans*, including in AVA neurons [80]. We found that *daf-2(e1370)* mutants exhibit decreased surface levels of GLR-1 and block the increase in surface GLR-1 observed in *unc-29/*AChR mutants (Fig 5). Future studies will be required to investigate whether DAF-2 acts specifically in AVA neurons to mediate the effects of INS-27 on GLR-1 trafficking. Interestingly, while *ins-27* mutants partially block the increase in surface GLR-1 triggered by *unc-29*/AChR mutants, *daf-2* mutants completely block the feedback pathway. These data suggest that one or more ILPs act together with INS-27 to mediate the feedback pathway.

In conclusion, our study identifies a neuropeptide secreted from muscle that regulates surface levels of AMPARs in upstream neurons, revealing a novel muscle-to-neuron signal that regulates AMPAR trafficking. Given the large number of secreted factors implicated in inter-tissue and inter-organ communication, including between muscle and the brain [19,29], we anticipate that there are other extracellular AMPAR-regulatory factors secreted from non-neuronal tissues that remain to be discovered.

## Materials and Methods

### Strain maintenance and genotyping

*C. elegans* strains were maintained at 20°C on standard Nematode Growth Medium (NGM) agar plates spotted with OP50 *E. coli* unless otherwise stated. Larval stage 4 (L4) hermaphrodites were used in all experiments unless otherwise specified. 3–4 L4 worms of healthy, normal-growing strains were transferred twice a week to new plates. Many *unc* strains grow slowly and have fewer progeny so propagation patterns were altered for these strains. *unc-54(e1301ts)* worms were maintained at the permissive temperature of 15°C to prevent muscle paralysis. *daf-2(e1370)* worms were also maintained at 15°C to prevent dauer formation [124]. Genotyping was performed using PCR and/or sequencing to detect deletion alleles or point mutations, plate phenotypes, fluorescence, or a combination of these approaches. See S1 Table for a list of strains used in this study. See S2 Table for the primer sequences used for PCR genotyping and sequencing.

### Molecular Biology

Plasmids were constructed with standard restriction enzyme cloning techniques unless otherwise stated. See S2 Table for primer sequences used for PCR to insert restriction sites for subcloning.

FJ#140 – *myo-3p::UNC-29::unc-54 3’UTR* was generated from KP#1288 (*myo-3p::UNC-29::GFP*)(gift from David Simon and Josh Kaplan). Not I flanked GFP was removed from KP#1288 using Not I to generate FJ#140.

FJ#146 – *myo-3p::UNC-31* was constructed by removing *pvf-1* from FJ#133 (*myo-3p::PVF-1*) [49] and inserting *unc-31* from KG121 (Addgene plasmid #110879 [125]) using Nhe I and Kpn I sites. Nhe I and Kpn I restriction sites were added to *unc-31* via PCR using the UNC-31-FWD (Nhe I) and UNC-31-REV (Kpn I) cloning primers.

FJ#147 – *myo-3p::INS-27* was constructed by removing *pvf-1::gfp* from FJ#150 (*myo-3p::PVF-1::GFP*) [49] and inserting *ins-27* cDNA using Nhe I and Not I sites. Flanking Nhe I and Not I sites were added to the 252 bp open reading frame of *ins-27* isolated from cDNA generated from N2 animals and PCR with the INS-27-FWD (Nhe I) and INS-27-REV (Not I) cloning primers.

FJ#148 – *myo-3p::INS-27::mCherry* was constructed by inserting mCherry (Not I flanked) into *myo-3p::INS-27* (FJ#147).

FJ#149 – *myo-3p::INS-27::VENUS* was constructed by removing mCherry from *myo-3p::INS-27::mCherry* (FJ#148) using Not I and inserting VENUS (Not I flanked) from pBALU6 (Addgene plasmid #69321 [126]. Flanking Not I sites were added to VENUS via PCR using VENUS FWD (Not I) and VENUS REV (Not I) cloning primers.

FJ#152 – *rig-3p::NLS::GFP::LacZ* was constructed by subcloning *rig-3p* from *rig-3p::mCherry* using Sph I and Bam HI restriction enzymes and inserting the promoter into pPD96.04 (a promoterless NLS::GFP::LacZ construct, Addgene plasmid #1502, from Fire Vectors).

FJ#153 - *myo-3p::HisCl1* was constructed by Gibson assembly from templates FJ#150 (*myo-3p::PVF-1::GFP*) and NP#403 (Gift from Cori Bargmann) [70]. The HisCl1 coding region was amplified from NP#403 by PCR and inserted by Gibson assembly into the vector backbone of FJ#150 containing *myo-3p*. Primers used for Gibson assembly are listed in S2 Table. FJ#153 was injected into N2 worms at 10ng/µL with *unc-122p::GFP* at 25ng/µL and GeneRuler 1Kb Plus DNA Ladder (Thermo Scientific #SM1331) at 65ng/µL to make *pzEx509*.

### Temperature-shift experiments

For temperature shift experiments, early stage L4 worms were picked to new NGM plates and placed in an incubator set to 30°C (restrictive temperature) for the indicated duration. Although previous studies reported 25°C [127] as the restrictive temperature for *unc-54(e1301ts)* and >25°C for *twk-18(cn110ts)* [69], we used 30°C as the restrictive temperature for both alleles because we found that this temperature induced paralysis more consistently.

### Histamine-HisCl1 experiments

*akIs201* animals with or without the *myo-3p::HisCl1* transgene were exposed to NGM plates containing 10mM Histamine (SigmaH7250-5G) dissolved in molecular grade water and sterile filtered, as previously described [70]. At 30 min, 1hr, 2hr and 4hr time points, animals were imaged for SEP and mCherry fluorescence as described below.

### Eyelash nose touch assay

The nose touch assay was carried out as previously described [128]. Briefly, young adult worms were picked to NGM plates spotted with diluted OP50. An eyelash attached to the end of a wooden stick was placed in the crawling path of the worm so that the worm’s nose collided perpendicularly with the eyelash to primarily stimulate ASH dendrites. A response was scored as a reversal if the worm initiated a backward movement a distance greater than the length of the nose to the posterior pharyngeal bulb. A total of ten trials were completed for each worm and the results are expressed as a percentage. All assays were performed with the experimenter blinded to the genotypes being assayed.

### Confocal imaging

For all imaging experiments, worms were immobilized on a glass coverslip for 5 minutes in a 5 µL drop of M9 Buffer containing 30 mg/mL BDM. A 2% agarose pad on a glass slide was inverted over the droplet to mount the worms on the slide. Images were captured using a Zeiss LSM800 confocal microscope with a 63X Plan Apochromat objective (Numerical Aperture 1.4). All imaging was performed on L4 stage worms.

#### SEP::mCherry::GLR-1 and SEP::mCherry::NMR-2 imaging.

mCherry and SEP fluorescence were imaged in three regions: the nerve ring, the anterior VNC, and the posterior VNC. The imaging settings that captured a 7.92 µm thick z-stack of the nerve ring are as follows: Acquisition area: 50.71 X 50.71 µm, 488nm laser: 11.00%, Gain: 740V, 561nm laser: 4.50%, Gain: 700V. The following imaging settings were used to capture both the anterior and posterior VNC. Z-stack: 9.75 µm thick, Acquisition area: 101.53 X 53.92 µm, 488nm laser: 11.00%, Gain 800V, 561nm laser: 7.50%, Gain: 700V. For *SEP::mCherry::NMR-2.* mCherry and SEP fluorescence were imaged in the posterior VNC. The same imaging settings as noted above for *SEP::mCherry::GLR-1* were used. For imaging of SEP::mCherry::GLR-1 in the *daf-2* experiments, worms were maintained at 15°C to prevent dauer formation in *daf-2(e1370)* mutants. To image L4 animals at 20°C, 20 hours before imaging, L2 animals were transferred to 20°C.

#### *rig-3p*::NLS::GFP::LacZ imaging.

GFP fluorescence in nuclei of AVA cell bodies in the head was acquired in a 14.7µm thick z-stack using the following imaging settings: Acquisition area: 33.80 X 33.80µm, 488nm laser: 0.3%, Gain 500V. DIC images were also captured to confirm the identity of the AVA cell bodies during imaging analysis.

#### INS-27::VENUS imaging.

Images of INS-27::VENUS accumulation in vesicles inside of anterior coelomocytes (marked with RFP (*unc-122p::RFP*)(Addgene)) were acquired using the following settings: Z-stack: 9.9µm thick, Acquisition area: 50.71 X 50.71µm, 488nm laser: 0.2%, Gain 500V, 561nm laser: 0.5%, Gain 500V.

### Image analysis

#### SEP::mCherry::GLR-1 and SEP::mCherry::NMR-2 image analysis.

SEP::mCherry::GLR-1 and SEP::mCherry::NMR-2 images were processed with Zeiss Zen Blue software. For all images, a single pixel filter was applied to both channels to reduce noise. Then an orthogonal maximum intensity projection was obtained from all slices of the z-stack containing the ventral nerve cord (VNC). A custom MATLAB script was built to analyze the total fluorescent intensity of a region of interest (ROI) for each fluorophore (SEP and mCherry). The script facilitates opening each image, prompts the user to draw an ROI surrounding the VNC or NR, sets a pixel threshold for each channel (SEP: 45, mCherry: 55) to reduce background noise, then sums the pixel values in the ROI to obtain the total fluorescent intensity of the ROI. These values are recorded before proceeding through all images within a given imaging session, recording all values in a summary table. Each value is normalized to the wild-type control (*akIs201* with no experimental manipulation) geometric mean for that imaging session. The script outputs an Excel file containing both the total raw fluorescent intensity values and the normalized values for both fluorophores of each VNC for all experimental genotypes and the control. Results from different imaging sessions were then manually pooled and statistical tests were performed. Graphs show individual data points; each data point corresponds to the ROI drawn around the VNC of one worm. SEP::mCherry::NMR-2 images were processed and analyzed as described above for SEP::mCherry::GLR-1 except for the pixel thresholds for each channel (SEP: 45, mCherry: 45).

Histamine treatment of *akIs201* decreased SEP fluorescence at each time point starting at 30 minutes (SEP fluorescence of *akIs201* treated with Histamine, normalized to the 0-hour timepoint (Geometric mean, lower 95% CI- upper 95% CI): 0hr: 1.0, 0.7-1.4; 30 min: 0.6, 0.3-1.3; 1h: 0.8, 0.4-1.4; 2h: 0.5, 0.3-0.8; 4h: 1.2, 0.6-2.3). Therefore, the SEP fluorescence of Histamine treated animals expressing *myo-3p::HisCl* was normalized to the SEP fluorescence of Histamine treated WT animals (*akIs201*) for each respective time point and graphed in S4 Fig.

#### INS-27::VENUS in coelomocyte image analysis.

Maximal projections of each channel were obtained in ImageJ. The total fluorescent intensity was recorded from ROIs that were drawn around each vesicle in the coelomocyte. The fluorescent intensity of the 5 brightest vesicles were averaged to obtain a single value for each coelomocyte. One or two anterior coelomocytes were analyzed per worm. All values were normalized to the wild-type geometric mean from the corresponding imaging session before pooling across sessions. Individual data points on the graph correspond to single coelomocytes.

#### *rig-3p*::NLS::GFP::LacZ image analysis.

Maximum intensity projections for each image were obtained in ImageJ. An ROI was drawn around the cell body of AVA and the total fluorescent intensity of the ROI was recorded. Experimental genotypes were normalized to the wild-type average for the imaging session before pooling data across sessions.

### Statistics

All statistical analyses were performed in PRISM (GraphPad, v10.4.1). To determine which statistical analysis was appropriate for each data set, we used the built in Normality Test. When the Normality Test identified a greater likelihood of lognormal data and the data satisfy the requirements of lognormal data, we used the appropriate non-parametric statistical tests, and graphed the data to display the geometric means with error bars representing the upper and lower 95% confidence intervals (CIs), which are indicated in the figure legends. When the Normality Test identified a greater likelihood that the data set was normal (i.e., Fig 4E, which contains valid values equal to 0), we used the appropriate parametric statistical tests and graphed the data to display the arithmetic mean with error bars representing the standard error of the mean (SEM). To determine the statistical significance for the lognormal data in Figs 3D and 4G, we transformed each data point using the equation Y = log(Y) and then used a Two-way ANOVA with Sidak’s multiple comparison test.

## Supporting information

S1 Fig Effects of loss of cholinergic signaling on surface and total GLR levels in AVA neurons. **(A)** Representative images showing SEP fluorescence (surface GLR-1) in AVA processes in the posterior VNC in animals expressing a SEP::mCherry::GLR-1 integrated transgene (*akIs201*) in WT or *unc-38(e264)* mutant animals. **(B)** Quantification of SEP fluorescence (Surface GLR-1) for the strains shown in (A). The graph shows Geometric mean, lower 95% CI - upper 95% CI for WT (1.00, 0.84-1.19, n = 83) and *unc-38(e264)*(3.96, 3.37-4.64, n = 23) mutant animals. Values that differ significantly from WT (Kruskal-Wallis, Dunn’s multiple comparison test) are indicated as follows: ***p < 0.001. **(C)** Representative images showing mCherry fluorescence (total GLR-1) in AVA processes in the nerve ring (NR)(straightened) in animals expressing a SEP::mCherry::GLR-1 integrated transgene (*akIs201*) in WT or *unc-29(x29)* mutant animals. **(D)** Quantification of mCherry fluorescence (Normalized) for the strains shown in C. The graph shows Geometric mean, lower 95% CI - upper 95% CI for WT (1.00, 0.58-1.72, n = 10) and *unc-29(x29)*(2.02, 1.19-3.45, n = 9) mutant animals. Values that differ significantly from WT (Kolmogorov-Smirnov test) are indicated as follows: n.s., p > 0.05. **(E)** Representative images showing mCherry fluorescence (total GLR-1) in AVA processes in the anterior VNC in animals expressing a SEP::mCherry::GLR-1 integrated transgene (*akIs201*) in WT or *unc-29(x29)* mutants. **(F)** Quantification of mCherry fluorescence (Normalized) for the strains shown in E. The graph shows Geometric mean, lower 95% CI - upper 95% CI for WT (1.00, 0.84-1.19, n = 65) and *unc-29(x29)* mutant animals (1.23, 1.04-1.46, n = 64). Values that differ significantly from WT (Kolmogorov-Smirnov test) are indicated as follows: n.s., p > 0.05. **(G)** Representative images showing mCherry fluorescence (total GLR-1) in the AVA processes in the posterior VNC in animals expressing a SEP::mCherry::GLR-1 integrated transgene (*akIs201*) in WT, *unc-29(e1072),* or *unc-29(x29)* mutants, or *unc-29(x29)* mutants expressing WT *unc-29* cDNA under control of the *myo-3* promoter *(pzEx476: myo-3p::UNC-29 line #1* and *pzEx477: myo-3p::UNC-29 line #2*). **(H)** Quantification of mCherry fluorescence (Normalized) for the strains shown in G. The graph shows Geometric mean, lower 95% CI - upper 95% CI for WT (1.00, 0.90-1.11, n = 83), *unc-29(e1072)* (1.17, 0.97-1.39, n = 30), *unc-29(x29)* (1.54, 1.37-1.73, n = 66), *unc-29(x29); pzEx476 (myo-3p::UNC-29 line #1)*(0.69, 0.58-0.82, n = 51), and *unc-29(x29); pzEx477 (myo-3p::UNC-29 line #2)* (0.71, 0.61-0.83, n = 54). Values that differ significantly from WT (Kruskal-Wallis, Dunn’s multiple comparison test) are shown above each bar. Other comparisons are marked by brackets and are indicated as follows: n.s., p > 0.05, *p < 0.05, ***p < 0.001.
(PDF)

S2 Fig Loss of *unc-29*/AChR or *unc-54*/myosin does not affect surface levels of the NMDA receptor subunit NMR-2. **(A)** Diagram showing the *flp-18p*::SEP::mCherry::NMR-2 transgene. **(B)** Representative images showing SEP fluorescence (surface NMR-2) in AVA processes in the posterior VNC in animals expressing a SEP::mCherry::NMR-2 integrated transgene (*akIs237*) in WT or *unc-29(x29)* mutant animals or *unc-54(e1301ts)* mutants after 4h shift to the restrictive temperature. **(C)** Quantification of SEP fluorescence (Normalized) for the strains shown in B. The graph shows Geometric mean, lower 95% CI – upper 95% CI for WT (1.00, 0.80-1.25, n = 33), *unc-29(x29)* (1.35, 0.88-2.07, n = 27), and *unc-54(e1301ts)* 4-hrs TS (1.50, 1.21-1.86, n = 26). Values did not differ significantly from WT (Kruskal-Wallis, Dunn’s multiple comparison test) (n.s., p > 0.05).
(PDF)

S3 Fig Loss of *unc-29*/AChRs does not increase surface GLR-1 levels by regulating the *rig-3* promoter. Nuclear GFP fluorescence was measured in WT or *unc-29(x29)* mutant animals expressing a *rig-3* transcriptional reporter *pzIs46 (rig-3p::NLS::GFP::LacZ*). GFP fluorescence (Norm.) in AVA nuclei: WT (1.00, 0.91-1.10, n = 23) and *unc-29(x29)* (1.11, 1.02-1.21, n = 32). The graph shows Geometric mean, lower 95% CI - upper 95% CI. n.s., p > 0.05 (Kolmogorov-Smirnov test).
(PDF)

S4 Fig Acute inactivation of muscle using the Histamine-HCl1 chemical genetic system results in increased surface GLR-1 levels in AVA neurons. Graph showing SEP fluorescence in AVA processes in the posterior VNC in animals expressing a SEP::mCherry::GLR-1 integrated transgene (*akIs201*) and HisCl1 in muscle (*myo-3p::HisCl1*). Quantification of SEP fluorescence: HisCl1 + Histamine normalized to control treated with Histamine at each time point. The graph shows Geometric mean, lower 95% CI – upper 95% CI for *myo-3p::HisCl1:* 0 hrs (1.59, 1.13-2.25, n = 45), 0.5 hrs (2.61, 1.37-4.95, n = 21), 1 hr (4.82, 2.98-7.79, n = 19), 2 hrs (3.78, 2.26-6.35, n = 33), 4 hrs (6.78, 3.83-12.00, n = 16). ns., p > 0.05, **p < 0.01, ***p < 0.001 (Kruskal-Wallis, Dunn’s multiple comparison test).
(PDF)

S5 Fig *unc-31* rescue experiments. The Graph shows the quantification of SEP fluorescence in AVA processes in the posterior VNC in animals expressing a SEP::mCherry::GLR-1 integrated transgene (*akIs201*) in the genotypes indicated on the x-axis. Geometric mean, lower 95% CI – upper 95% CI for WT (1.00, 0.85-1.17, n = 77), *unc-31(e928)* (0.18, 0.14-0.23, n = 76), *unc-31(e928); pzEx479(myo-3p::UNC-31)* (0.27, 0.19-0.40, n = 28), and *unc-31(e928); pzEx498(rab-3p::UNC-31)* (2.51, 1.79-3.52, n = 20). Values that differ significantly from WT (Kruskal-Wallis test, Dunn’s multiple comparison test) are shown above each bar. Other comparisons are marked by brackets. ns., p > 0.05, *p < 0.05, ***p < 0.001.
(PDF)

S1 Table List of Strains used in this study. (DOCX)

S2 Table List of Primers used for molecular biology and genotyping. (DOCX)

S3 Table Raw data values used to calculate the normalized values in the Figures. (XLSX)

S4 Table Normalized values and geometric means used to generate the graphs. (XLSX)
