# Supplementary figures and images for: The insulin-like peptide INS-27 mediates a muscle-to-neuron feedback signal coupling muscle activity with AMPA receptor trafficking
Source: PLoS Genet. 2025 Jul 18;21(7):e1011786. doi: 10.1371/journal.pgen.1011786 (PMC12303390; doi:10.1371/journal.pgen.1011786)

# S1 Fig

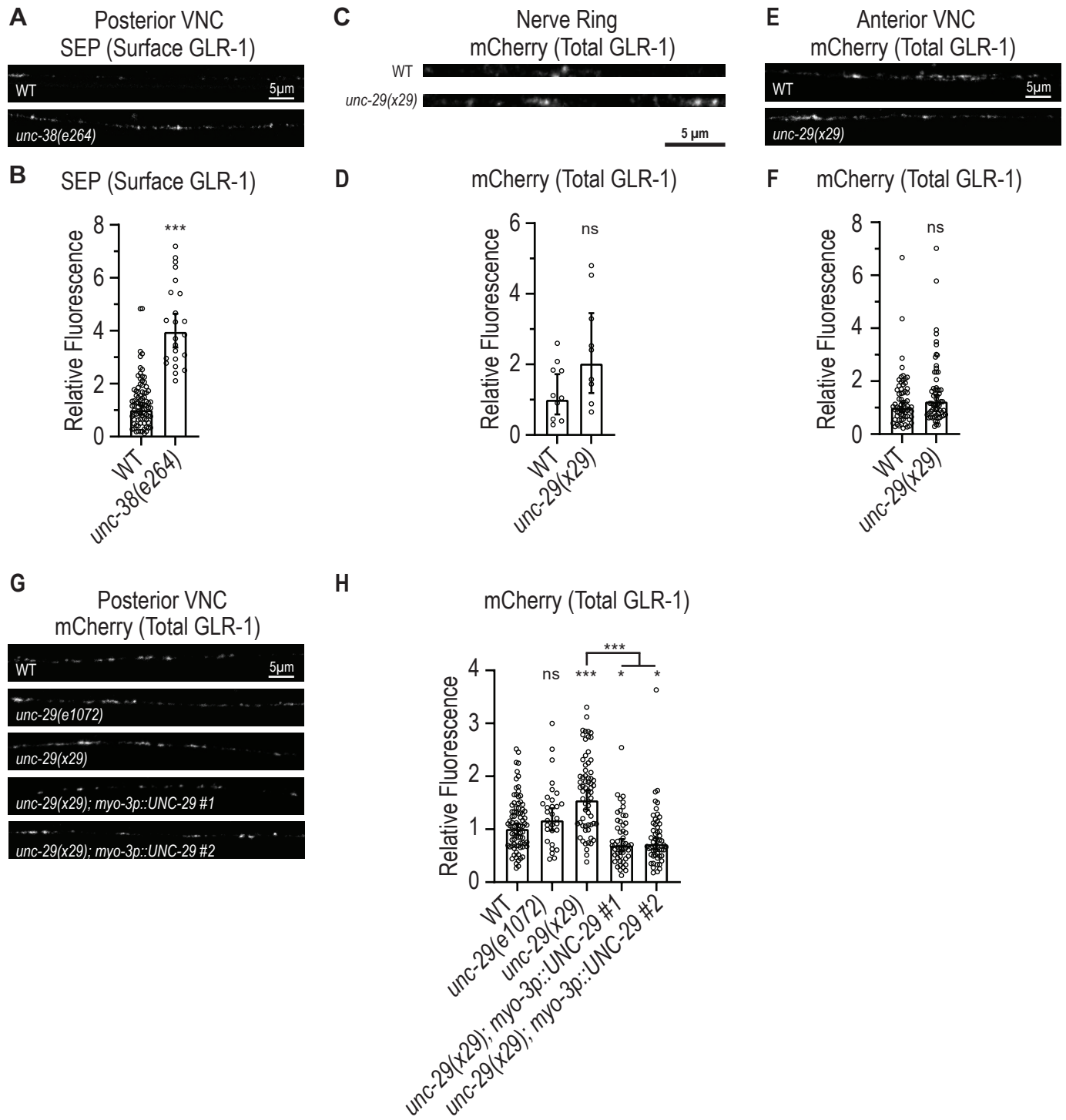

Supplement: S1 Fig — (A) Representative images showing SEP fluorescence (surface GLR-1) in AVA processes in the posterior VNC in animals expressing a SEP::mCherry::GLR-1 integrated transgene (akIs201) in WT or unc-38(e264) mutant animals. (B) Quantification of SEP fluorescence (Surface GLR-1) for the strains shown in (A). The graph shows Geometric mean, lower 95% CI - upper 95% CI for WT (1.00, 0.84-1.19, n = 83) and unc-38(e264)(3.96, 3.37-4.64, n = 23) mutant animals. Values that differ significantly from WT (Kruskal-Wallis, Dunn’s multiple comparison test) are indicated as follows: ***p < 0.001. (C) Representative images showing mCherry fluorescence (total GLR-1) in AVA processes in the nerve ring (NR)(straightened) in animals expressing a SEP::mCherry::GLR-1 integrated transgene (akIs201) in WT or unc-29(x29) mutant animals. (D) Quantification of mCherry fluorescence (Normalized) for the strains shown in C. The graph shows Geometric mean, lower 95% CI - upper 95% CI for WT (1.00, 0.58-1.72, n = 10) and unc-29(x29)(2.02, 1.19-3.45, n = 9) mutant animals. Values that differ significantly from WT (Kolmogorov-Smirnov test) are indicated as follows: n.s., p > 0.05. (E) Representative images showing mCherry fluorescence (total GLR-1) in AVA processes in the anterior VNC in animals expressing a SEP::mCherry::GLR-1 integrated transgene (akIs201) in WT or unc-29(x29) mutants. (F) Quantification of mCherry fluorescence (Normalized) for the strains shown in E. The graph shows Geometric mean, lower 95% CI - upper 95% CI for WT (1.00, 0.84-1.19, n = 65) and unc-29(x29) mutant animals (1.23, 1.04-1.46, n = 64). Values that differ significantly from WT (Kolmogorov-Smirnov test) are indicated as follows: n.s., p > 0.05. (G) Representative images showing mCherry fluorescence (total GLR-1) in the AVA processes in the posterior VNC in animals expressing a SEP::mCherry::GLR-1 integrated transgene (akIs201) in WT, unc-29(e1072), or unc-29(x29) mutants, or unc-29(x29) mutants expressing WT unc-29 cDNA un [file pgen.1011786.s001.pdf]

S2 Fig

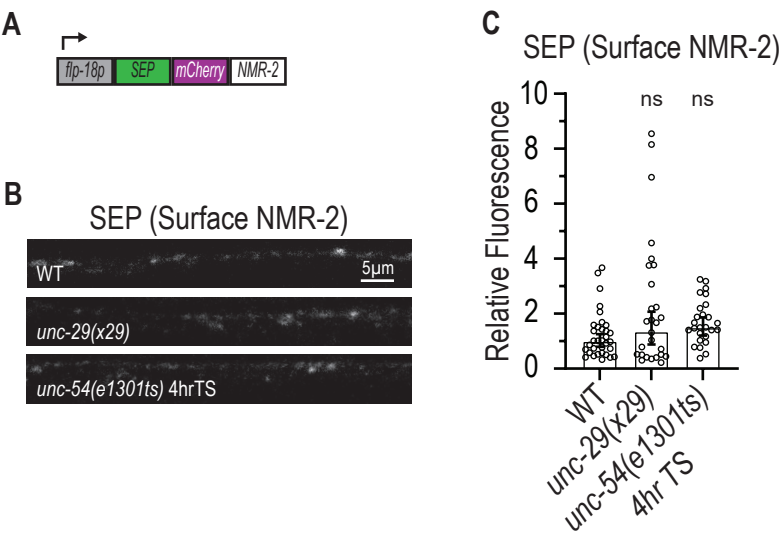

Supplement: S2 Fig — (A) Diagram showing the flp-18p::SEP::mCherry::NMR-2 transgene. (B) Representative images showing SEP fluorescence (surface NMR-2) in AVA processes in the posterior VNC in animals expressing a SEP::mCherry::NMR-2 integrated transgene (akIs237) in WT or unc-29(x29) mutant animals or unc-54(e1301ts) mutants after 4h shift to the restrictive temperature. (C) Quantification of SEP fluorescence (Normalized) for the strains shown in B. The graph shows Geometric mean, lower 95% CI – upper 95% CI for WT (1.00, 0.80-1.25, n = 33), unc-29(x29) (1.35, 0.88-2.07, n = 27), and unc-54(e1301ts) 4-hrs TS (1.50, 1.21-1.86, n = 26). Values did not differ significantly from WT (Kruskal-Wallis, Dunn’s multiple comparison test) (n.s., p > 0.05). (PDF) [file pgen.1011786.s002.pdf]

S3 Fig

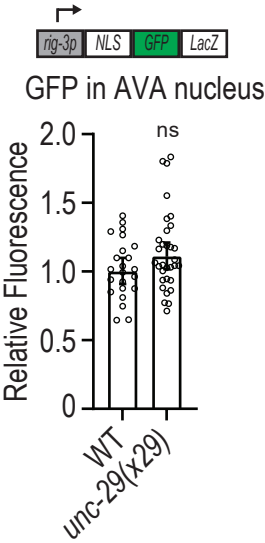

Supplement: S3 Fig — Nuclear GFP fluorescence was measured in WT or unc-29(x29) mutant animals expressing a rig-3 transcriptional reporter pzIs46 (rig-3p::NLS::GFP::LacZ). GFP fluorescence (Norm.) in AVA nuclei: WT (1.00, 0.91-1.10, n = 23) and unc-29(x29) (1.11, 1.02-1.21, n = 32). The graph shows Geometric mean, lower 95% CI - upper 95% CI. n.s., p > 0.05 (Kolmogorov-Smirnov test). (PDF) [file pgen.1011786.s003.pdf]

S4 Fig

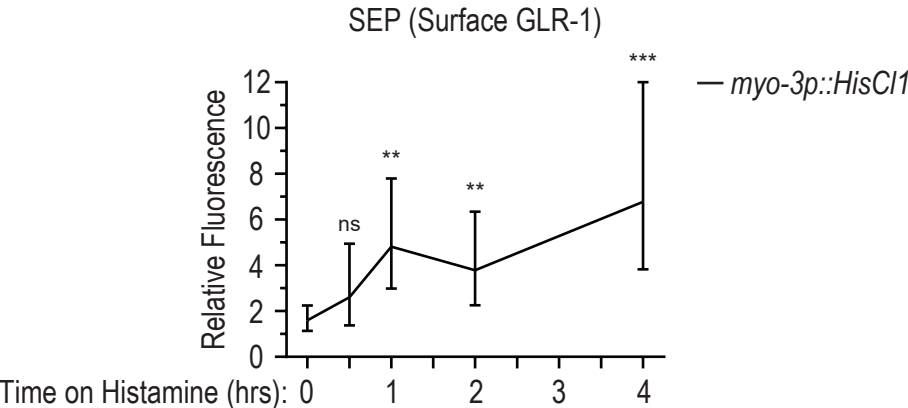

Supplement: S4 Fig — Graph showing SEP fluorescence in AVA processes in the posterior VNC in animals expressing a SEP::mCherry::GLR-1 integrated transgene (akIs201) and HisCl1 in muscle (myo-3p::HisCl1). Quantification of SEP fluorescence: HisCl1 + Histamine normalized to control treated with Histamine at each time point. The graph shows Geometric mean, lower 95% CI – upper 95% CI for myo-3p::HisCl1: 0 hrs (1.59, 1.13-2.25, n = 45), 0.5 hrs (2.61, 1.37-4.95, n = 21), 1 hr (4.82, 2.98-7.79, n = 19), 2 hrs (3.78, 2.26-6.35, n = 33), 4 hrs (6.78, 3.83-12.00, n = 16). ns., p > 0.05, **p < 0.01, ***p < 0.001 (Kruskal-Wallis, Dunn’s multiple comparison test). (PDF) [file pgen.1011786.s004.pdf]

S5 Fig

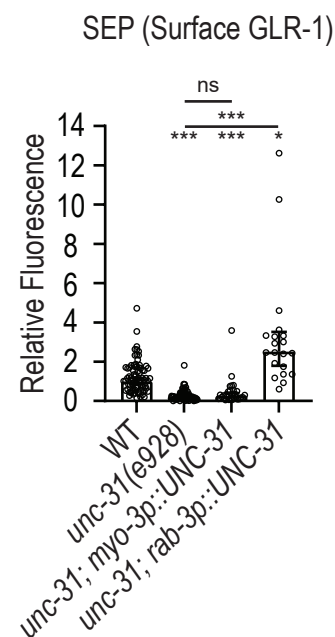

Supplement: S5 Fig — The Graph shows the quantification of SEP fluorescence in AVA processes in the posterior VNC in animals expressing a SEP::mCherry::GLR-1 integrated transgene (akIs201) in the genotypes indicated on the x-axis. Geometric mean, lower 95% CI – upper 95% CI for WT (1.00, 0.85-1.17, n = 77), unc-31(e928) (0.18, 0.14-0.23, n = 76), unc-31(e928); pzEx479(myo-3p::UNC-31) (0.27, 0.19-0.40, n = 28), and unc-31(e928); pzEx498(rab-3p::UNC-31) (2.51, 1.79-3.52, n = 20). Values that differ significantly from WT (Kruskal-Wallis test, Dunn’s multiple comparison test) are shown above each bar. Other comparisons are marked by brackets. ns., p > 0.05, *p < 0.05, ***p < 0.001. (PDF) [file pgen.1011786.s005.pdf]
