# Supplementary material for: The insulin-like peptide INS-27 mediates a muscle-to-neuron feedback signal coupling muscle activity with AMPA receptor trafficking
Source: PLoS Genet. 2025 Jul 18;21(7):e1011786. doi: 10.1371/journal.pgen.1011786 (PMC12303390; doi:10.1371/journal.pgen.1011786)
Supplement: S1 Table — (DOCX) [file pgen.1011786.s006.docx]

**S1 Table – List of strains used in this study.**

| **Strain** | **Genotype** |
| --- | --- |
| N2 | Wild type (Bristol) |
| FJ1499 | *akIs201[rig-3p::SEP::mCherry::GLR-1]* V (gift from Frederic Hoerndli and Villu Maricq) |
| FJ1664 | *akIs201 V*; *unc-29(x29)* *I* |
| FJ1676 | *akIs201 V;* *unc-29(e1072) I* |
| FJ1888 | *akIs201 V*; *unc-38(e264) I* |
| FJ1865 | *akIs201 V; unc-29(x29) I; pzEx476 [myo-3p::UNC-29 + unc-122p::GFP line 1] line #1* |
| FJ1866 | *akIs201 V; unc-29(x29) I; pzEx477 [myo-3p::UNC-29 + unc-122p::GFP line 3] line #2* |
| FJ1852 | *pzIs46 [rig-3p::NLS::GFP::LacZ + unc-122p::RFP]* |
| FJ1854 | *pzIs46; unc-29(x29)* |
| FJ1867 | *akIs201 V; unc-54(e1301) I* |
| FJ1768 | *akIs201 V; twk-18(cn110) X* |
| FJ1751 | *akIs201 V; unc-31(e928) IV* |
| FJ1877 | *akIs201 V; unc-31(e928); pzEx479[myo-3p::UNC-31 + unc-122p::RFP]* |
| FJ2036 | *akIs201 V; unc-31(e928); pzEx498[rab-3p::UNC-31 + unc-122p::RFP]* |
| FJ1761 | *akIs201 V; unc-31(e928) IV; unc-29(x29) I* |
| FJ1951 | *akIs201 V; unc-31(e928) IV; unc-29(x29) I; pzEx479[myo-3p::UNC-31 + unc-122p::RFP]* |
| FJ2031 | *akIs201 V; unc-31(e928) IV; unc-29(x29) I; pzEx498[rab-3p::UNC-31 + unc-122p::RFP]* |
| FJ1941 | *akIs201 V; twk-18(cn110) X; unc-31(e928) IV* |
| FJ1661 | *akIs201 V; unc-29(x29) I; pvf-1(ev763) III* |
| KP0004 | *glr-1(n2461)*4 III* |
| FJ2013 | *ins-27(ok2474)*5 I* |
| FJ2014 | *ins-27(ok2474) I; pzEx484[myo-3p::INS-27 + unc-122p::GFP]*5* |
| FJ1966 | *akIs201 V; ins-27(ok2474)*4 I* |
| FJ1968 | *akIs201 V; ins-27(ok2474) I; pzEx484*4* |
| FJ1949 | *akIs201 V; pzEx484*4* |
| FJ1950 | *akIs201 V; ins-27(ok2474),unc-29(x29) I* |
| FJ1967 | *akIs201 V; ins-27(ok2474),unc-29(x29) I; pzEx484* |
| FJ1946 | *akIs201 V; twk-18(cn110) X; ins-27(ok2474) I* |
| FJ1935 | *pzEx488[myo-3p::INS-27::VENUS + unc-122p::RFP]* |
| FJ1955 | *pzEx488; unc-29(x29) I* |
| FJ1954 | *pzEx488; unc-31(e928) IV* |
| FJ1960 | *pzEx488; unc-29(x29) I; unc-31(e928) IV* |
| FJ1986 | *pzEx488; twk-18(cn110)* |
| VM10512 | *akIs237 [flp-18p::SEP::mCherry::NMR-2]* (gift from Villu Maricq) |
| FJ2076 | *akIs237; unc-29(x29) I* |
| FJ2082 | *akIs237; unc-54(e1301) I* |
| FJ2032 | *akIs201 V; daf-2(e1370) IV* |
| FJ2033 | *akIs201 V; unc-29(e1072) I; daf-2(e1370) IV* |
| FJ2080 | *akIs201 V; pzEx509 [myo-3p::HisCl1 + unc-122p::GFP]* |
