# Supplementary material for: The insulin-like peptide INS-27 mediates a muscle-to-neuron feedback signal coupling muscle activity with AMPA receptor trafficking
Source: PLoS Genet. 2025 Jul 18;21(7):e1011786. doi: 10.1371/journal.pgen.1011786 (PMC12303390; doi:10.1371/journal.pgen.1011786)
Supplement: S2 Table — (DOCX) [file pgen.1011786.s007.docx]

**S2 Table – List of primers used in this study.**

| **Genotyping Primers** | **Sequence** |
| --- | --- |
| unc-31(e928) F | GAAAAATCCATTGCCCGTGGTG |
| unc-31(e928) R | GATAGCTGAGATATGAGCTGGC |
| unc-31(e928) R del | CCCTTTAAGTGCGCCTTCAGG |
| e928 F2 (genomic) | GAGGTCGTGAGAGAAAGCTAG |
| e928indelR2genomic | GCACCCATAGCAACTCCAATTC |
| unc-29(x29) F | GGGACACCACTTTTTGGCGGTG |
| unc-29(x29) R | GGAGGCCTTTCCCAGGTGTT |
| x29 seq | GGTGCCCGACAAAGTTGTATTG |
| twk-18(cn110) F | AAGTCCACCAGGGTTGTGAGC |
| twk-18(cn110) R | CGCGCACAAATTTGGCACTGC |
| ins-27(ok2474) OL | TGTTCAAAACGCACTTGGAG |
| ins-27(ok2474) OR | TCAAAGCCCCATAACTTTGC |
| ins-27(ok2474) IL | ATATTACCGCTGGTTGCTCC |
| ins-27(ok2474) IR | CAAGCTTCAGCGCATAAACA |
| daf-2(e1370) F | CAACCAGCGATGGTTGTGATGG |
| daf-2(e1370) R | CCGAATCACTCTGAACCTCGACG |
|  |  |
| **Cloning Primers** | **Sequence** |
| UNC-31-FWD (Nhe I) | GATCGCTAGCAAAAATGTTAGGAGCAAGTAGTAGTGAAG |
| UNC-31-REV (Kpn I) | GATCGGTACCTTAGGCGGCCGCATGTTTTCGTATACCTTCTTG |
| INS-27 FWD (Nhe I) | GATCGCTAGCAAAAATGAAATTCTTCCGCTTAATCTTGC |
| INS-27 REV (Not I) | GATCGGCGGCCGCAGATGAGAAAGTTGGGTCTTCAGATA |
| VENUS FWD (Not I) | GTCAGATCGCGGCCGCCGTGAGCAAGGGCGAGGAGCTGTTC |
| VENUS Rev (Not I) | GATCGGTACCTCAGGCGGCCGCCTTGTACAGCTCGTCCATGCCGAG |
| Pmyo3Gibson F | GGTATTGATATCTGAGCTCCGC |
| Pmyo3Gibson R | CTAGTGGTCGTGGGTTTGATG |
| HisClGibson F | CATCAAACCCACGACCACTAGAAAAATGCAAAGCCCAACTAGC |
| HisCl Gibson R | GCGGAGCTCAGATATCAATACCTCATAGGAACGTTGTCCA |
| HisClInt R | GCAACCAGTCGACATCCAAT |
